# 4-Coumarate 3-hydroxylase in the lignin biosynthesis pathway is a cytosolic ascorbate peroxidase

**DOI:** 10.1038/s41467-019-10082-7

**Published:** 2019-04-30

**Authors:** Jaime Barros, Luis Escamilla-Trevino, Luhua Song, Xiaolan Rao, Juan Carlos Serrani-Yarce, Maite Docampo Palacios, Nancy Engle, Feroza K. Choudhury, Timothy J. Tschaplinski, Barney J. Venables, Ron Mittler, Richard A. Dixon

**Affiliations:** 1 BioDiscovery Institute, University of North Texas, Denton, TX 76203 United States; 2 Department of Biological Sciences, University of North Texas, Denton, TX 76203 USA; 3 Center for Bioenergy Innovation (CBI), Oak Ridge National Laboratory, Oak Ridge, TN 37831 USA; 4 BioEnergy Science Center (BESC), Oak Ridge National Laboratory, Oak Ridge, TN 37831 USA

**Keywords:** Enzymes, Cell wall, Secondary metabolism

## Abstract

Lignin biosynthesis is evolutionarily conserved among higher plants and features a critical 3-hydroxylation reaction involving phenolic esters. However, increasing evidence questions the involvement of a single pathway to lignin formation in vascular plants. Here we describe an enzyme catalyzing the direct 3-hydroxylation of 4-coumarate to caffeate in lignin biosynthesis as a bifunctional peroxidase that oxidizes both ascorbate and 4-coumarate at comparable rates. A combination of biochemical and genetic evidence in the model plants *Brachypodium distachyon* and *Arabidopsis thaliana* supports a role for this coumarate 3-hydroxylase (C3H) in the early steps of lignin biosynthesis. The subsequent efficient *O*-methylation of caffeate to ferulate in grasses is substantiated by in vivo biochemical assays. Our results identify C3H as the only non-membrane bound hydroxylase in the lignin pathway and revise the currently accepted models of lignin biosynthesis, suggesting new gene targets to improve forage and bioenergy crops.

## Abstract (web summary)

Lignin biosynthesis in higher plants relies upon a 3-hydroxylation reaction that can occur via shikimate esters of 4-coumarate. Here, Barros et al. define an alternative biosynthetic pathway via cytosolic ascorbate peroxidase that can catalyze direct 3-hydroxylation of 4-coumarate.

## Introduction

**Fig. 1:**
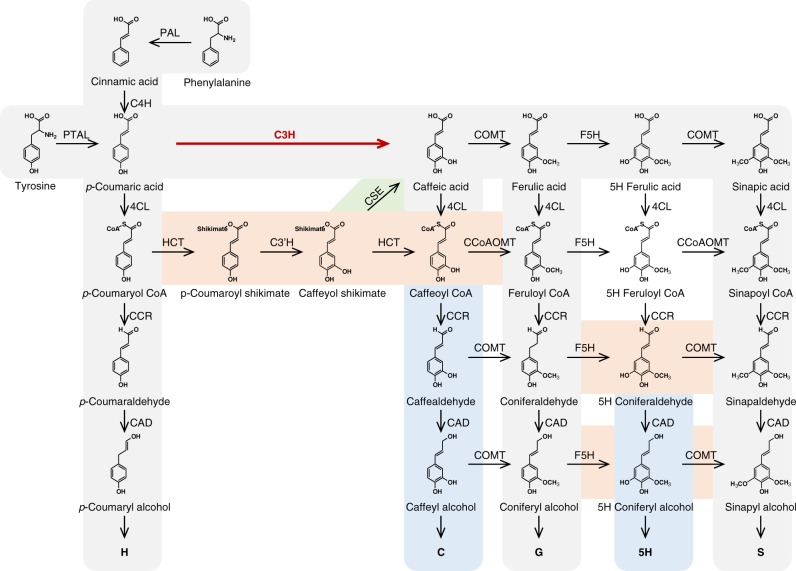
The lignin biosynthetic pathway highlighting the 4-coumarate 3-hydroxylase (C3H) reaction characterized in this study. Shadings illustrate key findings in the current understanding of lignin biosynthesis in plants. Model of lignin biosynthesis as understood in the 80 s (in gray), shikimate shunt and substrate preferences of P450 enzymes discovered in the 90 s (in brown), subsequently identified 5H- and C-lignin monomers (in blue), and most recent step discovered (in green). PAL l-phenylalanine ammonia-lyase, PTAL bifunctional l-phenylalanine/l-tyrosine ammonia-lyase, C4H cinnamate 4-hydroxylase, C3H 4-coumarate 3-hydroxylase, COMT caffeate/5-hydroxyferulate 3-O-methyltransferase, F5H ferulate 5-hydroxylase/coniferaldehyde 5-hydroxylase, 4CL 4-hydroxycinnamate:CoA ligase, HCT 4-hydroxycinnamoyl CoA:shikimate/quinate hydroxycinnamoyltransferase, C3’H 4-coumaroyl shikimate/quinate 3’-hydroxylase, CSE caffeoyl shikimate esterase, CCoAOMT caffeoyl CoA 3-O-methyltransferase, CCR cinnamoyl CoA reductase, CAD, cinnamyl alcohol dehydrogenase

Because of its importance for plant vascular function^1^ and stress responses^2^, and the economics of the food, paper, pulp, and biorefining industries^3^, the biosynthesis of the cell wall polymer lignin is one of the most intensively studied areas of plant biochemistry. Lignin is predominantly composed of three monomers known as monolignols (*p*-coumaryl, coniferyl, and sinapyl alcohols), which are polymerized in the apoplast of vascular and fiber cells^1^ leading to the formation of the *p*-hydroxyphenyl (H), guaiacyl (G), and syringyl (S) units of lignin, respectively. The 3-hydroxylation of the 4-coumarate moiety diverts flux from H toward G and S lignin, and this reaction is now generally accepted to occur at the level of the shikimate ester of 4-coumarate^4,5^ (Fig. 1). However, the facts that some grass species lack orthologs of caffeoyl shikimate esterase (*CSE*)^6,7^ and that  reduced expression of some ester pathway enzymes has less than expected to no phenotypic effect in grasses^5,8^, along with paradoxical results of metabolomics and labeling studies in *Arabidopsis*^9^, support the existence of an alternative pathway to the currently accepted route involving phenolic esters. A suggestion that the coumarate 3-hydroxylase (C3H) reaction is catalyzed by a complex of two membrane-bound cytochrome P450 enzymes, the coumaroyl shikimate 3′-hydroxylase and cinnamate 4-hydroxylase (C3′H/C4H)^10^ (Fig. 1), is not supported by recent localization studies^11^. Instead, early biochemical studies suggested that C3H was a soluble phenolase that required molecular oxygen and a reducing agent such as ascorbate to oxidize a wide variety of substrates, including free 4-coumarate^12^. Although enzymes with similar properties were subsequently reported in monocot and dicot plants^13,14^, in some cases with high specificity for 4-coumarate^15^, C3H remains genetically and functionally uncharacterized.

In this study, we identify and characterize a bifunctional cytosolic ascorbate peroxidase as C3H, the missing link in the conventional phenylpropanoid pathway involving free phenolic acids.

## Results

### Identification and characterization of C3H

**Fig. 2:**
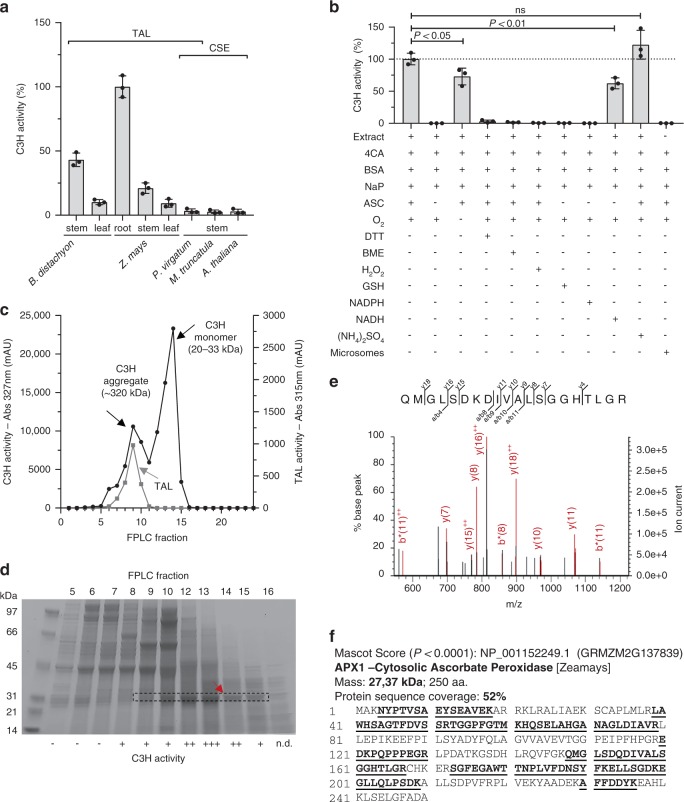
Characterization and identification of maize C3H. **a** C3H activity in several tissues from different plant species: *Brachypodium distachyon* 30-day-old stems and leaves, *Zea mays* 30-day-old in vitro grown roots, *Zea mays* 40-day-old field grown stems and leaves, *Panicum virgatum* mature tillers, *Medicago truncatula* young stems, and *Arabidopsis thaliana* 30-day-old stems. TAL and CSE denote the presence in the genomes of these plants of bifunctional l-phenylalanine/l-tyrosine ammonia-lyase and orthologs of caffeoyl shikimate esterase genes, respectively. **b** C3H activity in crude protein extracts from maize roots tested under the conditions indicated. Abbreviations and concentrations: 4CA 4-coumarate (1 mM), BSA bovine serum albumin (75 μg/ml), NaP sodium phosphate buffer pH 6 (75 mM), ASC ascorbate (4 mM), O_2_ incubation with oxygen (+) or under N_2_ atmosphere (–), DTT dithiothreitol (100 mM), BME 2-mercaptoethanol (250 mM), H_2_O_2_ hydrogen peroxide (0.03%), CAT catalase (11 KU ml^–1^), GSH glutathione (2 mM), NADPH reduced nicotinamide adenine dinucleotide phosphate (2 mM), NADH reduced nicotinamide adenine dinucleotide (2 mM), (NH_4_)_2_SO_4_ ammonium sulfate (1 mM). **c** C3H (black line) and TAL (gray line) activity in different FPLC fractions prepared from maize root extracts. **d** SDS–PAGE gel used to separate the proteins of the fractions obtained from gel filtration chromatography. The main band from fraction 14 (red arrow) was excised and subjected to trypsin digestion and peptide mass mapping by ESI-MS/MS. The dashed box outlines the main protein band correlated with the C3H activity (+, –, n.d., high, low, or not detected, respectively). The full uncropped gel is provided as a Source Data file. **e** MS/MS fragmentation of one peptide matching the C3H protein sequence. The y-, and b- ions are indicated, ++ denotes doubly charged ions and * denotes loss of NH_3_ group. **f** Mascot summary obtained after using the monoisotopic masses of the tryptic digest to match against NCBI plant databases. Error bars indicate mean ± SD, two-sided unpaired *t*-test, *n* = 3

A previously described protocol^13^ with minor modifications (Supplementary Fig. 1) was used to assay C3H activity in crude protein extracts prepared from tissues of several plant species, and the highest activities were found in maize and *Brachypodium* (Fig. 2a), both of which lack *CSE* orthologs^7^. The highly active maize root extract was subjected to further characterization (Fig. 2b). C3H activity was detected in the soluble but not the microsomal fraction. The activity was ascorbate dependent, completely inhibited by 2-mercaptoethanol, dithiothreitol and reduced glutathione, and decreased by 24% when incubated under N_2_. Addition of exogenous hydrogen peroxide completely abolished activity in crude extracts by causing depletion of ascorbate. NADH, but not NADPH, could partially replace ascorbate as co-factor. Using calibrated gel filtration chromatography, the molecular mass of the native maize root C3H was estimated to be 20–33 kDa (Fig. 2c). The presence of an earlier peak co-eluting at approximately 320 kDa with tyrosine ammonia-lyase (TAL) activity suggested that maize C3H may form part of a protein complex with other lignin pathway enzymes. The major band correlating with the C3H activity in the main peak of the partially purified C3H was subjected to sodium dodecyl sulphate -polyacrylamide gel electrophoresis (SDS–PAGE) (Fig. 2d) followed by in-gel digestion and peptide mass fingerprinting (Fig. 2e). The band showed the expected size of C3H, and the sequence was matched to a 27.37 kDa cytosolic ascorbate peroxidase (APX1, GRMZM2G137839), with 10 peptides covering about 52% of the maize sequence (Fig. 2f).

In *Arabidopsis*, the APX family is represented by eight members, three cytosolic, three microsomal, and two chloroplastic^16^. Using a phylogenetic approach, we identified putative C3H orthologs in a wide range of plant species (Supplementary Fig. 2). Purified and hemin reconstituted recombinant C3H from *Arabidopsis* and *Brachypodium* catalyzed both APX and C3H reactions in vitro with similar overall catalytic efficiencies (Supplementary Table 1). Recombinant C3H also hydroxylated l-tyrosine to form l-DOPA, but did not hydroxylate the other monolignol pathway intermediates 4-coumaroyl shikimate, 4-coumaroyl CoA, 4-coumaraldehyde, or 4-coumaryl alcohol under the conditions tested. Other cytosolic APXs can also oxidize aromatic substrates^17^, with reported binding sites at the δ-heme edge distinct from the ascorbate-binding site at the γ-heme edge^18,19^. Modeling the structure of *Brachypodium* C3H using the published crystal structure of soybean cytosolic APX^19^ confirmed that 4-coumarate could bind at the δ-edge side of the heme cofactor (Supplementary Fig. 3a, b). Multiple protein sequence alignments suggest that the main residues involved in catalysis are the same in monocot (grass) and dicot C3Hs (Supplementary Fig. 3c). The hydroxylation mechanism of C3H links the biosynthesis of phenylpropanoids with the detoxification of hydrogen peroxide (Supplementary Fig. 3d), and *C3H* is co-expressed with genes of the glutathione-ascorbate cycle, most lignin pathway genes, and other genes involved in cell wall biosynthesis and response to stress in *Arabidopsis* (Supplementary Fig. 4). Interrogation of publicly available microarray data from lignifying switchgrass (*Panicum virgatum*) cell cultures^20^ showed that C3H expression is negatively associated with expression of the “esters pathway” enzymes C3′H and CSE (Supplementary Fig. 5a). Further, consistent with involvement in lignin biosynthesis, antibodies raised against C3H detected the protein primarily in lignifying vascular tissue (Supplementary Fig. 6).

### Phenotypes of a *Brachypodium c3h* mutant

**Fig. 3:**
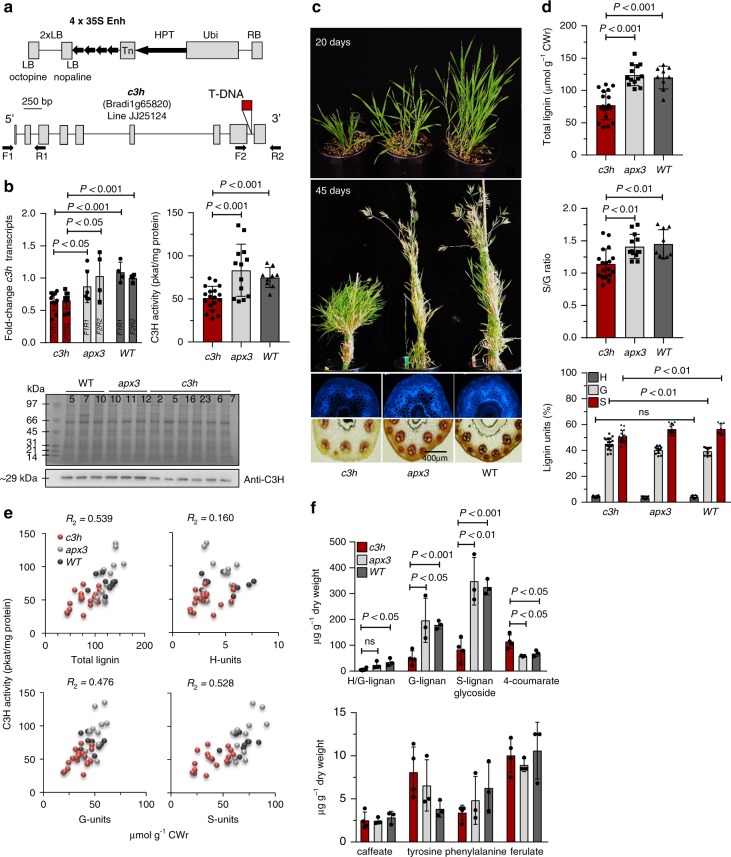
Phenotypic characterization of *Brachypodium c3h* mutants. **a** Activation tagging construct pJJ2LBA and diagram of the T-DNA insertion in line JJ25124 (http://jgi.doe.gov). **b** Relative expression by qPCR, extractable activity and protein level by immunoblotting in *c3h* mutants compared with wild-type and T-DNA control line JJ22251 (*apx3*). The antibodies raised against C3H showed no cross-reactivity and detect a band of 29.5 kDa^38^. Line numbers are indicated in each lane. The full uncropped gel and blot shown are provided as a Source Data file. **c** Growth phenotype and transverse stem sections (UV-autofluorescence and phloroglucinol-HCl staining) of *c3h* mutants and *apx3* and wild-type controls. **d** Total lignin (upper panel), S/G ratio (middle panel), and relative monolignol composition (lower panel) determined by thioacidolysis in *c3h* mutants and *apx3* and wild-type controls. **e** Correlation plots for C3H activity with total lignin amount and individual lignin monomers. **f** Metabolite concentrations in mature stems of *c3h* mutants compared with *apx3* and wild-type controls. Lignans were recognized from their fragmentation patterns. H/G lignan is 14.58 267 297 hydroxyphenyl guaiacyl lignan; G-lignan is 15.88 297 411 323 guaiacyl lignan, and S-lignan is 16.14 327 361 239 syringyl lignan glycoside (the first number is the retention time in min and the others are key mass-to-charge ratios, m/z). CWr cell wall residue. Error bars indicate mean ± SD, two-sided unpaired *t*-test. The *R*squared value (*R*^2^) was calculated from the linear regression model using Excel. Data points for all biological replicates are shown

To provide genetic evidence for a role of C3H in lignification, we first searched for *Brachypodium* mutants available from the JGI collection (https://jgi.doe.gov)^21^. Line JJ25124 was identified as an activation tagged line with the T-DNA insertion in the last intron, 121-bp upstream of the stop codon of *C3H* (Bradi1g65820) (Fig. 3a). The activation tagged line JJ16575, which has the T-DNA insertion in the last exon of the *APX3* gene (Bradi3g42340) encoding a microsomal APX was used as T-DNA control. Relative *C3H* transcript levels in the *c3h* lines were 30–40% lower than in the controls (Fig. 3b), and the extractable C3H activity was reduced (~40%), associated with reduced C3H protein amount determined by immunoblotting (Fig. 3b). The most plausible explanation for C3H downregulation in the *c3h* lines is the reported high frequency of methylation of the quadruple 35S enhancer sequence leading to its transcriptional silencing in T-DNA-based tagging populations^22^. The *Brachypodium c3h* lines showed a stunted bushy growth phenotype, delayed senescence, and few or non-viable seed (Fig. 3c). The lignin levels determined either by lignin autofluorescence, phloroglucinol staining, thioacidolysis, or reaction with acetyl bromide were significantly reduced when compared with controls, mainly from a reduction in lignin in fiber cells (high in S lignin) (Fig. 3c, d and Supplementary Table 3). Reduction in C3H extractable activity correlated with reduced total and S and G lignin deposition, with the *c3h* lines grouped together when compared with controls (Fig. 3e). Furthermore, gas chromatography/mass spectrometry (GC/MS)-based metabolic profiling showed that stems of the *c3h* mutants exhibited accumulation of 4-coumarate and depletion of H, G, and S lignans, but similar levels of caffeate, ferulate, tyrosine, and phenylalanine as compared with control plants (Fig. 3f).

### Phenotypes of *Arabidopsis c3h* mutants

**Fig. 4:**
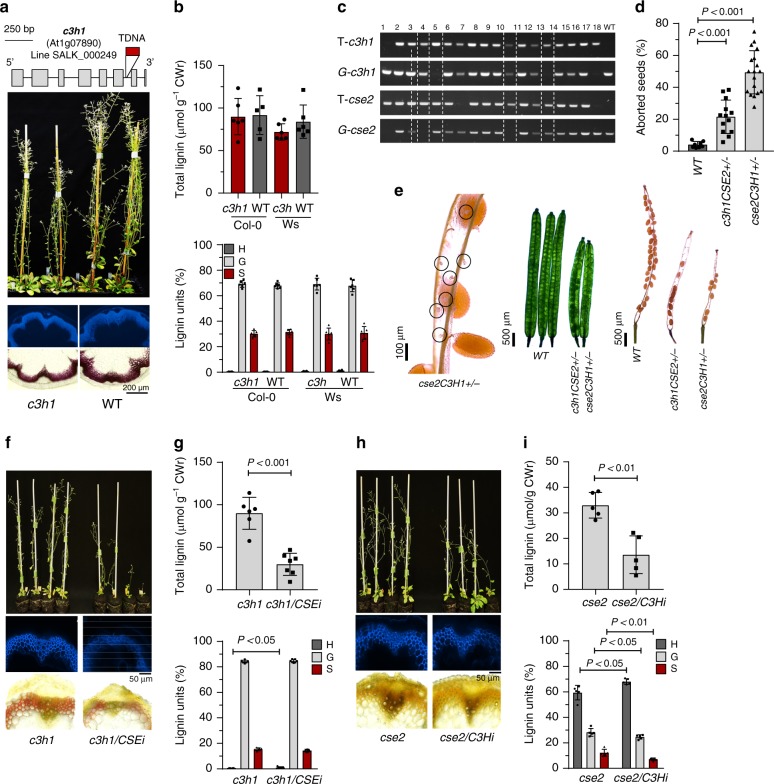
Phenotypic characterization of *Arabidopsis c3h1* mutants. **a** Position of the T-DNA insertion, mature *Arabidopsis* plants and transverse stem sections (UV-autofluorescence and phloroglucinol-HCl staining) of *c3h1* mutants and wild-type controls. **b** Lignin levels (upper panel) and relative monolignol composition (lower panel) determined by thioacidolysis of *c3h* mutants in both Col-0 and Wassilewskija (Ws) backgrounds. **c** Screening for *Arabidopsis c3h1/cse2* double mutants. T- and G- are T-DNA and gene specific primers used for genotyping. No *c3h1/cse2* double mutants were obtained from over 300 F2 plants screened, and so *cse2C3H1+/–* (i.e. homozygous for *cse2* and heterozygous for *c3h1*; lanes 4, and 11) and *c3h1CSE2+/–* (i.e., homozygous for *c3h1* and heterozygous for *cse2;* lanes 5 and 14) were subsequently generated and their lignin content and composition estimated (Supplementary Fig. 8). The full uncropped gels are provided as a Source Data file. **d** Aborted seeds in *c3h1CSE2+/–* and *cse2C3H1+/–* mutant lines determined in 8 to 18 individual siliques (segregation ratios estimated in Supplementary Table 2). **e** Comparison of the self-fertilized F3 seeds of mutants and WT plants by light microscopy in young (middle panel) and mature (right panel) siliques. The left panel shows a magnification of the aborted ovules (circles) in *cse2C3H1+/–* mutants. **f** Visual phenotype and transverse stem sections (UV-autofluorescence and Mäule staining) of *c3h1/CSE*-RNAi lines and *c3h1* controls. **g** Total lignin amount and composition of *c3h1/CSE*-RNAi lines and *c3h1* controls. **h** Visual phenotype and transverse stem sections (UV-autofluorescence and Mäule staining) of *cse2/C3H*-RNAi lines and *cse2* controls. **i** Total lignin amount and composition of *cse2/C3H*-RNAi lines and *cse2* controls. Mäule staining protocol was used in **f** and **h** to assess lignin distribution and composition. CWr cell wall residue. Error bars indicate mean ± SD, two-sided unpaired *t*-test. Data points for all biological replicates are shown

To investigate whether C3H plays a role in lignification in the dicot *Arabidopsis*, we first studied the SALK_000249 mutant line that has a T-DNA insertion in intron 7 of the *C3H* gene (At1g07890), 160-bp upstream of the stop codon (Fig. 4a). This line displays residual *APX1* transcript levels (15–30%) and APX activity (30%), but undetectable levels of C3H/APX1 protein as determined previously by immunoblotting^23,24^. *APX*-deficient mutant lines have been widely characterized in relation to the role of APXs in H_2_O_2_ detoxification and multiple abiotic stresses in both monocots and dicots^25–27^. The *Arabidopsis c3h1/apx1* mutant showed similar lignin autofluorescence, phloroglucinol staining, and lignin thioacidolysis yield as wild-type plants (Fig. 4a, b). However, metabolomic and published transcriptomic^23^ analyses of responses to light stress in the *c3h1/apx1* mutant revealed rapid reduction of caffeate levels, along with increased flavonoid glucoside levels and upregulated transcript levels of 4-coumarate: coenzyme A ligase (4CL) and flavonoid glucosyltransferases when compared with wild-type plants (Supplementary Fig. 7). These data support placement of C3H at the interface between 4-coumarate and caffeate in the initial steps of phenylpropanoid biosynthesis in *Arabidopsis*.

To further address the contribution of the acids pathway to lignification in *Arabidopsis*, we crossed the single *cse2* and *c3h1* mutants, generated their F_1_ progeny, and subsequently screened their F_2_ populations for *cse2:c3h1* double mutants. After genotyping >300 F_2_ plants, no double mutants could be recovered. We were only able to identify genotypes either homozygous for *c3h* and heterozygous for *cse* (*c3h1CSE2+/–*) or homozygous for *cse* and heterozygous for *c3h* (*cse2C3H1+/*) (Fig. 4c), which exhibited weakly reduced lignin levels (Supplementary Fig. 8a, b). In contrast to wild-type, the siliques of these c*3h1CSE2+/–* and *C3H1+/–cse2* mutants contained a higher proportion of aborted seeds, close to the expected 3:1 ratio for segregation of the double homozygote (Fig. 4d, e and Supplementary Table 2). To overcome the problem of the lethal phenotype of the double homozygote, we generated RNA interference (RNAi) lines to target each gene independently in the opposite mutant background (Supplementary Fig. 9a, b). Both *cse2* mutant/*C3H*-RNAi and *c3h1* mutant/*CSE*-RNAi lines were recovered and exhibited growth defects and reduced lignin deposition when compared with their respective T-DNA mutant only controls (Fig. 4f–i, and Supplementary Table 3).

Because the *c3h1/apx1* mutant retains residual C3H activity, we obtained all other available *c3h* mutant alleles from the Arabidopsis Biological Resource Center (ABRC), including SALK lines 088596 (*c3h2*), 095678C (*c3h4*), and 143111 (*c3h3*) with the T-DNA insertions in the last intron, 3′-UTR region and fourth exon, respectively. In agreement with a previous report^28^, no homozygous plants were found for *c3h3*, so heterozygous *c3h3+/–* plants were characterized along with homozygous *c3h1, c3h2, c3h4*, and wild-type controls (Supplementary Fig. 10). When compared with the *c3h1(apx1)* mutant, the other *c3h* mutant alleles showed downregulated *C3H* (10–30%) and *CSE* (30–45%) transcript levels, and increased anthocyanin content in leaves, increased numbers of xylem vessels, and reduced total stem lignin and S/G ratios (Supplementary Fig. 10a–d). These results are consistent with the phenotype observed above for the *c3h* lines in *Brachypodium*, and indicate that the homozygous *c3h3–/–* null mutation is lethal in *Arabidopsis* (Col-0).

### Subsequent *O-*methylation of phenolic acids/esters

**Fig. 5:**
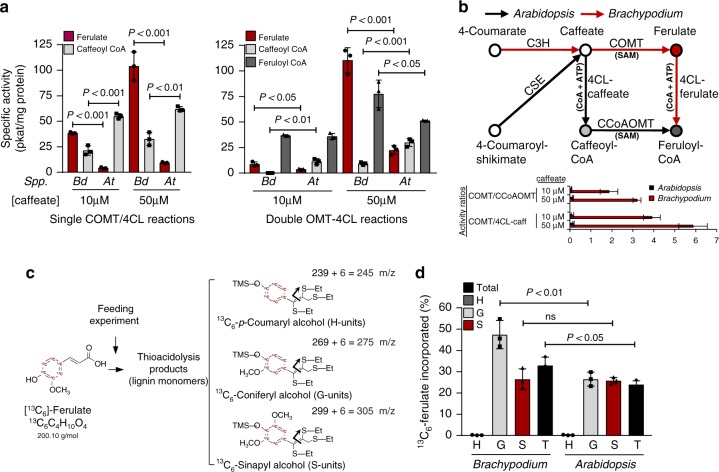
Biochemical assays to study the fate of caffeate in *Brachypodium* and *Arabidopsis*. **a** Specific activities of individual caffeate 3-*O*-methyltransferase (COMT) and 4-hydroxycinnamate:CoA ligase (4CL) reactions in crude stem protein extracts from 1-month-old *Brachypodium* and *Arabidopsis* plants, performed at 10 and 50 μM caffeate concentrations (left panel), and double reactions performed by co-incubating caffeate with both cofactors required to perform the CoA activation (CoA+ATP) and 3-methoxylation (S-adenosyl methionine, SAM) reactions (right panel). The final common product of both parallel activities is feruloyl CoA. **b** Scheme of the studied reactions including cofactors and showing the proposed most favored pathways in the model monocot *Brachypodium* (red arrows) and the model dicot *Arabidopsis* (black arrows). The bar plot displays the activity ratios of competing enzymatic activities for both species and substrate (caffeate) concentrations calculated from the data shown in panel **a**. **c** Labeling patterns of lignin monomers in isotopic feeding experiments (m/z, mass-to-charge ratio). **d** Percentage of ^13^C-labeled ferulate incorporated into different monolignols (H-, G-, and S-units) and total lignin (T) in roots of *Brachypodium* and *Arabidopsis* seedlings. C3H 4-coumarate 3-hydroxylase, CSE caffeoyl shikimate esterase, CCoAOMT caffeoyl CoA 3-*O*-methyltransferase. Error bars indicate mean ± SD, two-sided unpaired *t*-test. *n* = 3

Theoretically, caffeate can be either converted to caffeoyl CoA by 4CL, with subsequent *O*-methylation by caffeoyl CoA 3-*O*-methyltransferase (CCoAOMT), or first be methylated by caffeic acid 3-*O*-methyltransferase (COMT), with subsequent conversion to feruloyl CoA by 4CL (Fig. 1). To compare these alternatives in a grass and a dicot, we first determined the specific activities of COMT and 4CL for two different concentrations of caffeate in crude stem protein extracts from actively growing *Brachypodium* and *Arabidopsis* plants. The specific activity of COMT was much higher in extracts from *Brachypodium*, whereas 4CL activity was higher in *Arabidopsis* (Fig. 5a). When caffeate was co-incubated with both *S*-adenosyl-l-methionine (for the OMT reactions) and CoA+ATP (for the 4CL reactions), the main products accumulating in extracts from *Brachypodium* were ferulate and feruloyl CoA, whereas in extracts from *Arabidopsis* the main products were caffeoyl CoA and feruloyl CoA. Furthermore, the specific activity of maize root C3H towards 4-coumarate was higher than that of the competing reaction catalyzed by 4-hydroxycinnamoyl coenzyme A ligase (4CL) (Supplementary Table 4). The efficient methylation of caffeate by COMT in grasses might therefore drive the monolignol pathway through the C3H reaction (Fig. 5b). Consistent with the greater importance of the acids pathway in monocot grasses compared with dicots, we observed higher incorporation of ^13^C-labeled ferulate into the G-units of lignin in *Brachypodium* when compared with *Arabidopsis* (Fig. 5c, d). Furthermore, the lack of homologs of CSE, which is by-passed by the C3H reaction, seems to be phylogenetically associated with monocot plants (Supplementary Fig. 11). *Arabidopsis* CSE orthologs show ~80% identity among dicots, ~60% identity in non-commelinid monocots, and ~45% identity in most grasses. However, it remains unclear why rice and switchgrass retained CSE-like genes with 62 and 59% identity, respectively.

## Discussion

Our data demonstrate that a pathway to monolignols involving free phenolic acids is functional in both monocot and dicot model species. Notably, C3H is the only reported hydroxylase involved in phenylpropanoid biosynthesis that is not a membrane-bound cytochrome P450. Parallel pathways to the caffeoyl derivatives used as building blocks of S and G monolignols (Supplementary Fig. 12) may provide redundancy, and therefore robustness, to the early steps in the lignin pathway, especially as 4-coumaroyl CoA in the esters pathway is also the precursor for stress and developmentally induced flavonoid biosynthesis. These findings revise the current model of the monolignol pathway in plants to include a cytosolic route that, in grasses that possess TAL activity, by-passes both membrane-associated cytochrome P450 reactions involved in H and G lignin synthesis, and place the 3-*O*-methylation of monolignol precursors by COMT^29^ at the level of caffeic acid. This “soluble” pathway may be associated with detoxification of stress-induced reactive oxygen species and link the basic metabolic process of lignin biosynthesis with different stress responses, plant defense, and acclimation pathways. Further experimental evidence under multiple stress conditions will be necessary to better understand the cross-talk between phenylpropanoid metabolism and oxidative stress in plant cells.

Because of its bifunctionality, it is difficult to differentiate genetically the two activities of C3H/APX as causative of the lignin phenotypes recorded here. However, it should be noted that C3H is now the third monolignol pathway enzyme with an additional function associated with reactive oxygen metabolism. CSE is also a lysophospholipase that is induced by hydrogen peroxide and assists in phospholipid repair under stress conditions^6,30^, and CCR acts as an effector of RAC1, a GTPase involved in generation of active oxygen species during defense in rice^31^. The evolutionary implications of these associations remain to be explored.

## Methods

### Enzyme activity assays

The crude protein extracts and reaction mixtures used to assay 4-C3H activity were prepared using a modified protocol described previously^12^. Crude ammonium sulfate extracts were prepared by grinding in liquid N_2_ and homogenizing 2 g of fresh plant tissues in 6 ml of 50 mM sodium phosphate buffer pH 6 in the presence of 1 g polyvinylpolypyrrolidone (PVPP) and 1 g of 0.5 mm acid-washed glass beads. Samples were incubated overnight in a tube rotator at 4 °C. After filtration through four layers of Miracloth (Millipore) and centrifugation for 1 min at 500 *g* to remove the PVPP and glass beads, ammonium sulfate was added to a final concentration of 0.5 g ml^−1^. After centrifugation, the pellet was resuspended in one-half the original volume in 50 mM phosphate buffer pH 6. The homogenates were assayed directly. The reaction mixtures were optimized to a total volume of 100 µl containing 10 µl of 10 mM 4-coumarate, 7.5 µl of 10 mg ml^−1^ bovine serum albumin (BSA), 20 µl of 20 mM l-ascorbate, 7.5 µl of 1 M sodium phosphate buffer, 15 µl of the protein crude extracts equilibrated a 0.7 µg µl^−1^, and was incubated for 2 h at 30 °C with shaking and stopped with 10 µl of acetic acid. Incubating the reaction with phosphate buffer pH > 8 and preparing the crude protein extracts with antioxidants such as dithiothreitol or 2-mercaptoethanol or the stock solutions with dimethylsulfoxide completely inhibited C3H activity. Stock solutions were made with 50 mM phosphate buffer pH 6 and 4-coumarate was dissolved by sonication and heat (10 min at 96 °C) with shaking.

Previously described protocols were used to assay TAL^1^, COMT, and 4CL activity^5^. Briefly, 0.5–1 g of ground stem tissue from actively growing 1-month-old *Brachypodium* and *Arabidopsis* plants was resuspended in 2.7 ml of extraction buffer (100 mM Tris-Cl pH 7.5, 10% glycerol, 1 mM phenylmethylsulfonyl fluoride, and 0.5 mM dithiothreitol) in the presence of 0.1 g of PVPP. The suspension was incubated in a tube rotator at 4 °C for 45 min. The supernatant was recovered after centrifugation (12,000 × *g* for 5 min) and desalted using a PD-10 column (GE Healthcare) according to the manufacturer’s instructions. The protein concentrations were estimated with the Bio-Rad protein assay. In order to test COMT-4CL and 4CL-CCoAOMT reactions simultaneously, caffeic acid was co-incubated with *S*-adenosyl-l-methionine, coenzyme A and ATP. The reaction mixture contained ~10 µg of crude protein extract, 60 mM sodium phosphate buffer pH 7.2, 3 mM MgCl_2_, 2.5 mM ATP, 0.5 mM coenzyme A, 0.5 mM *S*-adenosyl-l-methionine, and 10 or 50 µM caffeate in a final volume of 100 µl. The reactions were incubated at 30 °C for 20 min, then stopped with 10 µl of acetic acid. Reaction products were analyzed by high performance liquid chromatography (HPLC) on a reversed phase C18 column (Spherisorb 5 µm ODS2 4.6 × 150 mm, Waters, Milford, MA) using a step gradient with 1% phosphoric acid water (solvent A) and acetonitrile (solvent B). Calibration curves were constructed with standards of the products.

### Protein purification and identification

All purification steps were carried out at 4 °C. Roots (23 g) from 30-day-old in vitro grown maize plants were ground in liquid nitrogen and homogenized in 125 ml of 50 mM sodium phosphate buffer pH 6, including 2.3 g of PVPP and the same amount of acid-washed glass beads. Homogenates were incubated overnight in a tube rotator. After Miracloth filtration and centrifugation as described above, the supernatant was concentrated 25-fold with a 10 kDa MW Amicon concentrator (Millipore), applied to a HiLoad 26/600 Superdex 200 (GE Healthcare) size exclusion chromatography column and eluted with 50 mM sodium phosphate buffer pH 6. Eluted fractions (1 ml) were combined consecutively in groups of four, Amicon concentrated 20-fold as above and the C3H activity determined. The fractions with high C3H activity were subjected to electrophoresis and the SDS–PAGE gel stained with Coomassie Blue Reagent (Bio-Rad). The main protein bands from the fraction with the highest C3H activity were excised with a clean scalpel for in-gel digestion. Gel pieces were transferred to microcentrifuge tubes and destained with 100 µl of 100 mM ammonium bicarbonate/acetonitrile (1:1, vol/vol) and incubated 30 min at room temperature with occasional vortexing. Acetonitrile (500 µl) was added and samples incubated for 10 min at room temperature with occasional vortexing until gel pieces become white and shrunken. The solution was removed and the samples were saturated with 50 µl of trypsin buffer (1.5 ml of 1 mM HCl to one 20 µg vial of trypsin (Sigma-Aldrich), divided into 100 µl aliquots and added 1.5 µl of 50 mM ammonium bicarbonate to the aliquot before use). Samples were incubated for 30 min at 4 °C, checked if more buffer was necessary to cover the pieces, and left for another 90 min at 4 °C. Then, 15 µl of 100 mM ammonium bicarbonate buffer were added to the samples to keep them wet during enzymatic cleavage. The digestion was performed by incubating the samples overnight at 37 °C in an air incubator. Samples were extracted with 100 µl of extraction buffer (5% formic acid/acetonitrile 1:2, vol/vol) for 15 min at 37 °C in a shaker incubator. The supernatant was collected into a PCR tube and dried down in a vacuum centrifuge for 30 min. The dried extracts were redissolved in 10 µl of 0.1% trifluoracetic acid, vortexed, and incubated for 3 min in a sonicator. Samples were centrifuged at 9600 *g* and the supernatant transferred to liquid chromatography/mass spectrometry (LC/MS) vials. Samples (8 μl) were injected into a capillary liquid chromatography-electrospray ionization-tandem mass spectrometer (LC-ESI-MS/MS) (Agilent Technologies) with a C18 reversed phase capillary column, 0.3 mm × 150 mm, (3.5 μm particles size). The LC separation consisted of solvent A: H_2_O/0.1% formic acid and solvent B: methanol /0.1% formic acid with the following gradient: 10 min 3% acetonitrile, 10 min 30%, 20 min 70%, 20 min 100% with a flow rate of 4 μl min^−1^ for 60 min. The peptides eluted were analyzed by ESI-MS/MS. Monoisotopic masses from the tryptic digests were used to identify the corresponding proteins by searching the plant databases in NCBI and SwissProt using the MASCOT search algorithm (http://www.matrixscience.com). The sensitivity of the method was determined using a commercial standard protein (BSA) digestate (Sigma-Aldrich) made by trypsin digestion at 1 pmol μl^−1^.

### Phylogenetic analysis

Protein sequences of C3H were BLAST searched using the NCBI GeneBank database. The phylogenetic analysis included protein sequences from six monocots (*Brachypodium distachyon, Oryza sativa, Setaria italica, Sorghum bicolor, Panicun virgatum* and *Zea mays*), five dicots (*Arabidopsis thaliana, Glycine max, Medicago truncatula, Pisum sativum* and *Populus trichocarpa*), one lycophyte (*Selaginella moellendorffii*), and one bryophyte used as outgroup (*Physcomitrella patens*). *P. virgatum* and *P. trichocarpa* protein sequences were obtained via Phytozome v12.0. Selected amino acid sequences with high homology were aligned using Geneious 10.0.9 software (Biomatters Ltd.) under ClustalW alignment and Blosum cost matrix. Phylogenetic trees were built by PhyML plugin using the JTT substitution model, 1000 bootstrap replicates optimized by topology, length and rate with the BEST network-network interface (NNI) and subtree pruning and re-grafting (SPR) topology search.

### Recombinant protein expression and kinetics

The full-length *Brachypodium* (Bradi1g65820) and *Arabidopsis* (At1g07890) *c3h* complementary DNA (cDNA) sequences were obtained from Phytozome v12.0 and amplified by RT-PCR (Phusion HiFi polymerase; New England BioLabs) from cDNA extracted from stem tissues of wild-type *B. distachyon* (Bd21–3) and *A. thaliana* (Col. 0) plants using the primer pairs (5′-CACCATGGCGAAGACCTACCCGAC-3′ and 5′-TCCGAACTGGGGTATGCTGAAGCTTAA-3′ for *Brachypodium* and 5′-CACCATGACGAAGAACTACCCAACCGT-3′ and 5′-CTGAGCTTGGGTTTGCTGATGCTTAA-3′ for *Arabidopsis*). Two different set of primers for At3g09640, a cytosolic ascorbate peroxidase 2 (APX2) gene homolog of *c3h*, were also tested but it was not possible to amplify the sequence by PCR from RNA of seedling, stem or leaf tissues. Based on the tissue-specific expression pattern in the *Arabidopsis* eFP Browser (http://bar.utoronto.ca/), APX2 is mainly expressed in mature pollen and at very low levels in other tissues when compared with APX1. Total RNA was isolated using Trizol reagent (Invitrogen) and first-strand cDNA was synthesized using the SuperScript III First-Strand System for RT-PCR Kit (Invitrogen) following the manufacturer’s instructions. The cDNAs were cloned into pENTR-D Topo and subsequently into pDEST17 vector by LR recombination reaction resulting in a 6xHis–C3H fusion construct. 6xHis-tagged C3H protein was expressed in *E. coli* strain Rosetta grown at 37 °C in Luria Bertani (LB) medium containing 0.1 mg ml^−1^ carbenicillin. After reaching an optical density at 600 nm of 0.7–0.9, protein expression was induced with 0.5 mM isopropyl β-D-1-thiogalactopyranoside (IPTG) and cells were grown at 16 °C for 18 h. Cells from 25 ml culture were harvested by centrifugation and resuspended in 2 ml of extraction buffer (50 mM Tris–HCl pH 8.0, 500 mM NaCl and 10 mM imidazole). All the following steps were carried out at 4 °C. Cell lysis was performed using an ultrasonic homogenizer (Model-120, Fisher Scientific). The lysates were recovered by centrifugation (16,000 *g*) for 20 min. Ni–NTA beads (Qiagen) were added and the suspension incubated at 4 °C for 30 min under constant inversion, and the unbound proteins were washed three times with 1 ml of extraction-washing buffer. Target proteins were eluted with 250 μl of elution solution (50 mM Tris–HCl buffer pH 8.0, 500 mM NaCl, and 250 mM imidazole) and their purity was verified by SDS–PAGE.

Preparation of reconstituted C3H was performed following procedures developed for soybean cytosolic APX with some modifications^32^. Both purified *Arabidopsis* and *Brachypodium* C3Hs were buffer exchanged in 50 mM sodium phosphate buffer at pH 6, and washed over a 10 kDa Amicon concentrator to a final volume of 4 ml. Bovine hemin (10 mg) was dissolved in 1 ml of 10 mM NaOH and brought to a final volume of 10 ml with 50 mM phosphate buffer pH 6. The hemin solution (1 ml) was slowly added in drops to the C3H solutions (4 ml) with gentle stirring. After 15 min at 4 °C with gentle shaking, the solutions were centrifuged at 12,000 *g* for 10 min to remove denatured reconstitution products. The concentration of the supernatants (reconstituted holoenzymes) were determined using the Bradford assay and directly used for kinetic assays. Enzyme kinetics were performed using 500 ng of the reconstituted protein in the optimized 100 µl reaction described above including 10 µl of 0.3% H_2_O_2_ solution and increasing concentrations (50–2000 µM) of the substrate 4-coumarate for C3H, ascorbate for APX and tyrosine for tyrosine hydroxylase enzymatic activities. Both tyrosine hydroxylase and C3H activities were determined by HPLC as describe above, whereas APX activity toward ascorbate (E290 = 2.8 mM^–1^ cm^–1^) was characterized spectrophotometrically using a Biotek SynergyMx microplate reader following^33^. Background oxidation was measured by combining substrate and H_2_O_2_ without reconstituted C3H. Since APX has been observed to display non-Michaelis–Menten kinetics^33,34^, the kinetic constants, *K*^n^ and *K*_cat_ were determined by using the solver function in Excel to find the best-fit parameters for the Hill equation: (*v* = *V*_max_[*S*]^n^/(*K*^n^ + [*S*]^n^), where *v* is the initial rate, *n* is a qualitative indication of the level of cooperativity, *K* is the substrate concentration at which the velocity is half-maximal, and *V*_max_ is the maximum velocity. When *n* = 1, the Hill equation reduces to the more usual Michaelis–Menten equation (*v* = *V*_max_/(1 + *K*_M_/[*S*])).

### Protein modeling

Comparative protein homology modeling was carried out with SWISS-MODEL to generate models for *Brachypodium* and *Arabidopsis* C3H using as a template the crystal structure of soybean APX/C3H (PDB ID: 1v0h). The structure models of co-factor heme and ligand ascorbate were obtained by superposition of the corresponding models onto the soybean APX/C3H structure. The substrates 4-coumarate was docked to the active site using the graphics program COOT (http://www2.mrc-lmb.cam.ac.uk/personal/pemsley/coot/) using as reference the structure of salicylhydroxamic acid bound to the soybean APX/C3H.

### Co-expression analyses

*Arabidopsis* C3H (At1g07890) was analyzed using the plant gene co-expression network tool PlaNet with default settings (http://aranet.mpimp-golm.mpg.de). The tool classifies genes according to their protein family annotation and compares gene vicinity networks two steps away (*N* = 2) from the query genes for re-occurring protein families^35^. Two additional online databases confirmed the co-expression of *Arabidopsis* C3H with most other lignin biosynthesis genes (Cressexpress, https://bitbucket.org/lorainelab/cressexpress and ATTEDII, http://atted.jp). *Brachypodium* C3H (Bradi1g65820) was not connected to any other gene in the PlaNet database and showed low expression specific to the first internode at 60 days after germination, therefore it was not possible to be draw the network of co-expressed genes. We therefore searched other publicly available co-expression datasets for monocots. Pearson’s correlation coefficients were obtained from microarrays conducted on cell cultures in which cell wall lignification was induced by application of brassinolide at 0, 6 h, 1, 3, and 7 days and on non-induced samples at 1 and 7 days as previously described^20^.

### Plant material and growth conditions

*Brachypodium* T-DNA line JJ25124 (IL000024891) was obtained from the JGI T-DNA collection (https://jgi.doe.gov/) and identified as an activation tagged line transformed with the pJJ2LBA vector and with the T-DNA insertion in the last intron with positive orientation, 121-bp upstream of the stop codon of the *c3h* gene Bradi1g65820. Line Bd21–3, the parent of the T-DNA mutant population, was used as the wild-type control. *Brachypodium* T-DNA line JJ22251 (IL000018556) was used as a T-DNA control transformed with the same pJJ2LBA activation tagging vector and positive T-DNA orientation, 632-bp downstream of the stop codon of a microsomal ascorbate peroxidase 3 (APX3, Bradi3g42340). T-DNA-positive F_2_ generation seeds received from the stock were grown and the individual T3 generation lines further characterized. *Brachypodium* seeds were sown in soil and vernalized at 4 °C for 3 days in the dark before moving to a growth chamber under 70% humidity and 16-h light/8-h dark at 24 °C and a light intensity of 100 µE m^–2^ s ^–1^. Plantlets were kept well-watered and covered with a dome for 7–10 days until they reached 6–8 cm tall. After this time, plantlets were transferred to half-gallon pots containing Metro-Mix 360 (Sun Gro Horticulture) and grown in the greenhouse for 12 weeks with 70% humidity and 14-h light at 25 °C, 10-h dark at 22 °C, supplemented with photosynthetically active radiation (PAR) lights when the range was out of 40–120 µE m^–2^ s^−1^ during the day period. Genotyping, C3H enzyme activity assays, immunoblotting, lignin histochemical staining, and determination of lignin amount and composition were performed on mature stem tissues from 12-week-old plants.

The *Arabidopsis c3h1* T-DNA insertion mutant line SALK_000249 was obtained from the Arabidopsis Biological Resource Center (ABRC) at Ohio State University. This line was previously characterized as a cytosolic ascorbate peroxidase 1 (APX1) mutant^24,36^ and contains a T-DNA insertion in the seventh intron of the gene At1g07890, 160-bp upstream of the stop codon. The genetic study in *Arabidopsis* was completed with the characterization of the mutant lines SALK_088596 (*c3h2*), SALK_095678C (*c3h3*), and SALK_143111 (*c3h4*) with the T-DNA insertions in the last intron, 3′-UTR region and fourth exon of the *c3h* gene At1g07890, respectively. The left primer 5′-CCACCCTGGAAGAGAGGTTAG-3′, right primer 5′-CAACGGATGTGTTCAAATCG-3′, and border primer 5′-ATTTTGCCGATTTCGGAAC-3′ were used to genotype plants for the *c3h* insertion. The *Arabidopsis* mutants deficient in *c3h* in the Wassilewskija (Ws) background were obtained as described by Pnueli et al.^23^. The *cse2* T-DNA insertion mutant line SALK_023077, also obtained from ABRC, was previously characterized as harboring an insertion in the gene encoding a CSE involved in lignin biosynthesis^3^ and annotated as a lysophospholipase 2 promoting tolerance to oxidative stress^30^. This line contains a T-DNA insertion in the second exon of the gene At1g52760, 621-bp downstream of the start codon. In this case, the border primer above, the left primer 5′-AAAACACATCAAAACGATGCC-3′, and the right primer 5′-CTCTCCTTGAATCAGCGAGTG-3′ were used for genotyping. We attempted to generate *cse2:c3h1* double mutants by crossing the respective single mutants. Ten plants of the F_1_ generation for the crosses made in each direction (*c3h1* × *cse2* and *cse2* × *c3h1*) were allowed to self-fertilize. The F_2_ generation was genotyped using the primers above and characterized for segregation ratios, seed phenotype, and lignin amount and composition using the single mutants and wild-type plants as controls. No *cse2:c3h1* double mutants were obtained from over 300 F_2_ plants screened. We therefore generated RNAi lines for each gene in both mutant backgrounds (*cse2* KO/*c3h1*-RNAi and *c3h1* KO/*cse2*-RNAi lines). To this end, 215 bp of the coding sequence of *cse* was amplified using the primer pairs 5′-CACCGTGATATGGAGAAAGTTG-3′ and 5′-TGAGGATATGAAACCAAGCAAG-3′. Similarly, 220 bp of the *c3h* coding sequence was amplified using the primers 5′-CACCCAAGCCCCAACCACCT-3′ and 5′-CGACAACTCTTACTTCAAGGAACTC-3′. The PCR product was introduced into pENTR-D Topo and then into pB7GWIWG2(I) vector (https://gateway.psb.ugent.be/) by LR recombination reaction. This binary vector provides tolerance to Basta (phosphinothricin) as a plant selectable marker, in contrast to the background SALK mutant *c3h* or *cse* transformed with the marker NTPII, which confers resistance to kanamycin. After confirmation by sequencing, the expression clones were introduced into *Agrobacterium tumefaciens* strain GV3101 (pMP90RK) by electroporation. Single *c3h1* and *cse2* mutant plants were transformed using floral dip, selected in soil with Basta (7.5 mg ml^–1^), genotyped by qRT-PCR and characterized for lignin phenotype. Seeds were vernalized as described above and transferred to a growth chamber for 8 weeks at a temperature of 22 °C, 70% humidity and a light intensity of 100 µE m^–2^ s^–1^, with a 16-h light/8-h dark photoperiod.

### Real time qPCR

Total RNA was extracted from mature stem tissue of *Brachypodium* T-DNA and *Arabidopsis* RNAi lines above using Trizol (Thermo Fisher). Total RNA concentration (3 μg) was quantified with a NanoDrop ND-1000 spectrophotometer (NanoDrop Technologies). To remove genomic DNA contamination, total RNA was treated with DNase for 30 min at 37 °C following the TURBO DNA-free™ Kit (Fisher scientific). First-strand cDNA was synthesized using the high-capacity cDNA reverse transcription kit (Applied Biosystems) according to the manufacturer’s protocol. The cDNA samples were diluted 20-fold and used as qRT-PCR templates. *Arabidopsis cse* (At1g52760) primers 5′-CTCTTTGGTTTGGCTGATACG-3′ (7) and 5′-CAGTAACTCTCTCATTGTTCCCAC-3′; *Arabidopsis c3h* (At1g07890) primers 5′-GCGAAGATTACAAGAAGGCTGT-3′ and 5′-CCACATTGCTCTTAGGTTGTTG-3′, and *Brachypodium c3h* (Bradi1g65820) primers 5′-CCTGATCCGAAGCTTTTACCTCCCAGTA-3′ and 5′-TACGATGAAGAAACCGGCCGAGCAG-3′ in the 5′-UTR region (F1-R1) or 5′-AGTGACCCTGTCTTCCGCCC-3′ and 5′-GCTGCTACTATGTGCGATCAGAGTGATC-3′ in the 3′-UTR region (F2-R2) were used for amplification. *Brachypodium* tubulin (Bradi1g10150) primers 5′-GCCTTTGTCCACTGGTATGT-3′ and 5′-AACTCTGCACCAACCTCTTC-3′ and *Arabidopsis* EF-1α (At5g60390) primers 5′-GAGCCCAAGTTTTTGAAGA-3′ and 5′-TAAACTGTTCTTCCAAGCTCCA-3′ were used as housekeeping genes.

### Western blotting and tissue printing

Crude mature stem protein extracts were prepared as above for determination of C3H activity and protein levels in *Brachypodium c3h* T-DNA lines and both T-DNA and wild-type controls. Samples were centrifuged and the protein concentration of the supernatant determined using the Bradford Assay (Bio-Rad). Proteins (~20 µg) were denatured in Laemmli buffer for direct resolution by SDS–PAGE and immunoblotting. For immunoblot analysis, a polyclonal antibody against C3H from pea (*Pisum sativum*) produced in rabbit^37^ and a polyclonal horseradish peroxidase conjugated goat anti-rabbit IgG (Sigma-Aldrich) were used as primary (1:2000) and the secondary antibody (1:50,000), respectively. Substrate detection was performed by chemiluminescence (ECL Western Blotting Substrate, Pierce) and film exposure. For tissue printing, stems from *Arabidopsis* and maize were cut into 1 mm thick sections with a razor blade and gently blotted dry with absorbent paper. The section was then pressed firmly onto nitrocellulose membrane for 15–20 s. Detection of anti-C3H IgG binding was carried out using goat anti-(rabbit IgG) serum conjugated to alkaline phosphatase and visualized using as substrate bromochloroindolyl phosphate/nitroblue tetrazolium.

### Histochemical staining and microscopy

Mature stems from *Brachypodium* and *Arabidopsis* plants above were cut and the bottom 2 cm embedded in 70% glycerol. Slices of 100 μm thickness were cut using a HM 650 V Vibrating Blade Microtome (Thermo Fisher) and stained with phloroglucinol-HCL (3% (w/v) phloroglucinol in ethanol: 12 N HCL in a 1:2 ratio). For Mäule staining, the cross-sections were immersed in 1% w/v KMnO_4_ for 5 min, rinsed twice with water, incubated in 12% HCl for 5 min, rinsed twice with water and observed after adding a few drops of 1.5% NaHCO_3_ solution. Light microscope images were taken with an EVOS™ XL Core Imaging System (Thermo Fisher). The images showing lignin autofluorescence were captured with an EVOS FL Cell Imaging System (Thermo Fisher) equipped with a DAPI led light cube (excitation: 357/44 nm; emission: 447/60 nm). Images for the seed phenotype characterization in *Arabidopsis* were taken with an AxioCam MRc 5 camera attached to a Zeiss Axio Zoom.V16 stereo zoom microscope.

### Metabolic profiling

For untargeted metabolite profiling in mature *Brachypodium* stem tissues, lyophilized, ground samples (~10 mg) were weighed into centrifuge tubes and extracted with 2 ml of 80% ethanol and 50 μl of internal standard sorbitol (1 mg ml^−1^). Samples were extracted overnight in a tube rotator at room temperature and then centrifuged at 1900 *g* for 20 min. The supernatant was decanted into scintillation vials and stored at −20 °C. A 1 ml aliquot was dried under a nitrogen stream, dissolved in 0.5 ml acetonitrile, and silylated to generate trimethylsilyl (TMS) derivatives. Samples (1 µl) were injected into a GC-MS Agilent 5975C inert XL operated in electron impact (EI; 70 eV) ionization mode as described previously^38^. Metabolite peaks were extracted using a characteristic mass-to-charge (m/z) ratio to minimize integration of co-eluting metabolites. The extracted peaks of known metabolites were scaled back to the total ion current (TIC) using previously calculated scaling factors. Peaks were quantified by area integration and normalized to the quantity of internal standard recovered, amount of sample extracted, derivatized, and injected. A user-created database (>2400 spectra) of EI fragmentation patterns of TMS-derivatized compounds and the Wiley Registry 10th Edition/NIST 2014 Mass Spectral Library were used to identify the metabolites in the samples. Unidentified metabolites were designated by their retention time and key m/z ratios.

For untargeted metabolite profiling in leaves of *Arabidopsis* during the light stress time-course experiment, plants grown in a peat pellet were exposed to 1000 µmol m^–2^ s^–1^ light intensity at 22 °C for a period of 0, 20, 60, and 90 s as previously described^39^. Five biological replicates, each composed of leaves pooled from at least 90 different 18- to 21-day-old plants in three technical repeats, were obtained for control Col-0 and *c3h* mutant and submitted to Metabolon, Inc. for UPLC-MS/MS analysis performed using a Waters ACQUITY UPLC and a Thermo-Finnigan LTQ mass spectrometer. Compounds were identified using libraries of purified standards. The intensity of the compounds identified were rescaled to set the median value equal to 1, the natural log was then calculated for comparison and statistical analysis.

### Measurement of lignin content and composition

The lowest 10 cm of the stem tissue of the *Brachypodium* and *Arabidopsis* plant material described above was harvested, separated from axillary leaves and stems and chopped in 2–5-mm pieces. Prior to thioacidolysis, samples were ball-milled under liquid nitrogen and the cell wall residues prepared by sequentially extracting ~300 mg of fresh tissue samples with consecutive washes of 100% methanol (once), chloroform/methanol (2:1) (twice), 100% methanol (once), and water (twice) at room temperature and lyophilized overnight. Ten milligrams of the freeze-dried samples were weighed into a 15 ml screw-cap vial with a Teflon-lined cap. Total lignin content and monomer composition were determined by thioacidolysis followed by GC–MS. Thioacidolysis reagent consisted of 2.5% boron trifluoride diethyl etherate (>47.5% BF3, Sigma-Aldrich) 10% ethanethiol (97%, Alfa Aesar), and 87.5% 1,4-dioxane by volume. Three milliliters of the thioacidolysis reagent was added to the vial containing the samples, capped tightly and heated to 100 °C for 4 h with occasional vortexing. The reaction was quenched by cooling on ice for 5 min. The pH of the reaction was brought to 3–4 by adding 4 ml of water and 0.85 ml of saturated sodium bicarbonate. Docosane in chloroform (50 µl at 3.02 mg ml^−1^) was added as an internal standard. Methylene chloride (3 ml) was added to the samples and the vials capped tightly and gently vortexed to ensure mixing. Solvent/water layers were separated by centrifugation at 500 *g* for 2 min. The lower organic layer was then transferred to new 15 ml screw-cap vials and two scoops of sodium sulfate added to the samples, which were allowed to sit overnight to absorb any remaining water. Three milliliters of the dry solvent were transferred to 4 ml screw-cap vials and dried under a nitrogen stream for 30 min at 40 °C. Once the samples were dried, 150 µl of a 1:1 pyridine:BSTFA (N,O-Bis(trimethylsilyl)trifluoroacetamide) solution was added, vortexed, and incubated for 30 min at 40 °C for derivatization prior to GC analysis. Samples were vortexed again, transferred to GC vials and run directly in a Hewlett–Packard 7890A gas chromatograph with a 5975C series mass selective detector (column: Agilent DB-5 ms, 60 m × 0.25 mm × 0.25 μm film thickness). The inlet, main GC oven and FID were held at 250 °C with a column flow of 0.6 ml min^−1^. The Low Thermal Mass (LTM) column temperature program began at 130 °C for 2 min and was ramped at 150 °C min^−1^ to 325 °C. Mass spectra were recorded in electron impact mode (70 eV) with 60–650 m/z scanning range. The ions studied were the following: 299 for the thioethylated syringyl monomer TMS (S-unit), 269 for the thioethylated coniferyl monomer TMS (G-unit), 239 for the thioethylated coumaryl monomer TMS (H-unit), and 57 for the internal standard docosane. The silylated monomers are unstable and were not stored at room temperature for more than 24 h prior to injection.

### Measurement of anthocyanins

Anthocyanin content was measured in *Arabidopsis c3h* mutants. Three leaves from four individual plants were ground in liquid nitrogen, and ~5 mg of dried material weighed, boiled for 3 min in extraction buffer (isopropanol:HCl:H_2_O = 18:1:81), and incubated in a rotor wheel at room temperature overnight. Samples were centrifuged (12,000 × *g* for 5 min), and the absorbance of the supernatants measured at 535 and 650 nm. Anthocyanin content was calculated as (A535 − A650)/g DW.

### Isotopic labeling experiments

^13^C_6_-labeled ferulate was synthesized following a previously described protocol^40^ with minor modifications. Knoevenagel–Doebner condensation was performed between ^13^C_6_-labeled vanillin (100.0 mg, 0.64 mmol) and malonic acid (133.3 mg, 1.28 mmol) in ethanol (1 ml) using piperidine (10 μl) as catalyst. The reaction took place under reflux at 78 °C for 8 h. The mixture was poured into ice, acidified with hydrochloric acid (pH ~ 4), filter under vacuum, washed with chilled water (10 ml), and re-crystallized in ethanol: water (1:1, v:v) to furnish 78% yield of ferulate. The purity of the labeled compound was assessed by LC/MS and ^1^H NMR. For the labeling experiments, wild-type *Brachypodium* and *Arabidopsis* plants were first grown in culture tubes containing ½ Gamborg’s B5 salt mixture and 0.5% Phytagel under continuous light conditions. After germination, seedlings were transferred to culture tubes containing 20 ml of the same medium fed with 0.1 mM ^13^C_6_-ferulate and harvested after three weeks. Control experiments using unlabeled ferulate were conducted in parallel. Separated roots were ground in liquid nitrogen and stored at –80 °C until use. Samples were extracted for thioacidolysis as described above and the percentage of label incorporated into the lignin monomers was calculated as follows:

% ^13^C incorporated = peak area labeled/(peak area labeled + peak area unlabeled) × 100.

Peak areas of the thioacidolysis products of lignin (H-, G-, and S-units) were identified, and the incorporation into total lignin estimated using the sum of the three individual peak areas.

### Statistics

Statistical analysis of the results was performed by unpaired, two-tailed *t*-test. A 95% confidence interval was used for statistics and *P* < 0.05 was considered significant. All statistical tests were performed using Prism software.

### Reporting summary

Further information on research design is available in the Nature Research Reporting Summary linked to this article.

## Supplementary information

Supplementary Information

Reporting Summary

Source Data

## Data Availability

The data that support the findings of this study are available within the paper and its Supplementary Information, or are available from the corresponding author upon reasonable request. The source data underlying Figs. 2a, b, d, 3b, d, f, 4b, c, d, g, i, 5a, d, Supplementary Figs. 5, 6, 7, 8a, b and Supplementary Tables 1, 2, 3 and 4 are provided as a Source Data file.
