# Supplementary material for: 4-Coumarate 3-hydroxylase in the lignin biosynthesis pathway is a cytosolic ascorbate peroxidase
Source: Nat Commun. 2019 Apr 30;10:1994. doi: 10.1038/s41467-019-10082-7 (PMC6491607; doi:10.1038/s41467-019-10082-7)
Supplement: Supplementary file 1 — Supplementary Information [file 41467_2019_10082_MOESM1_ESM.pdf]

Supplementary Information

4-Coumarate 3-hydroxylase in the lignin biosynthesis pathway is a cytosolic ascorbate peroxidase

Barros *et al*

a

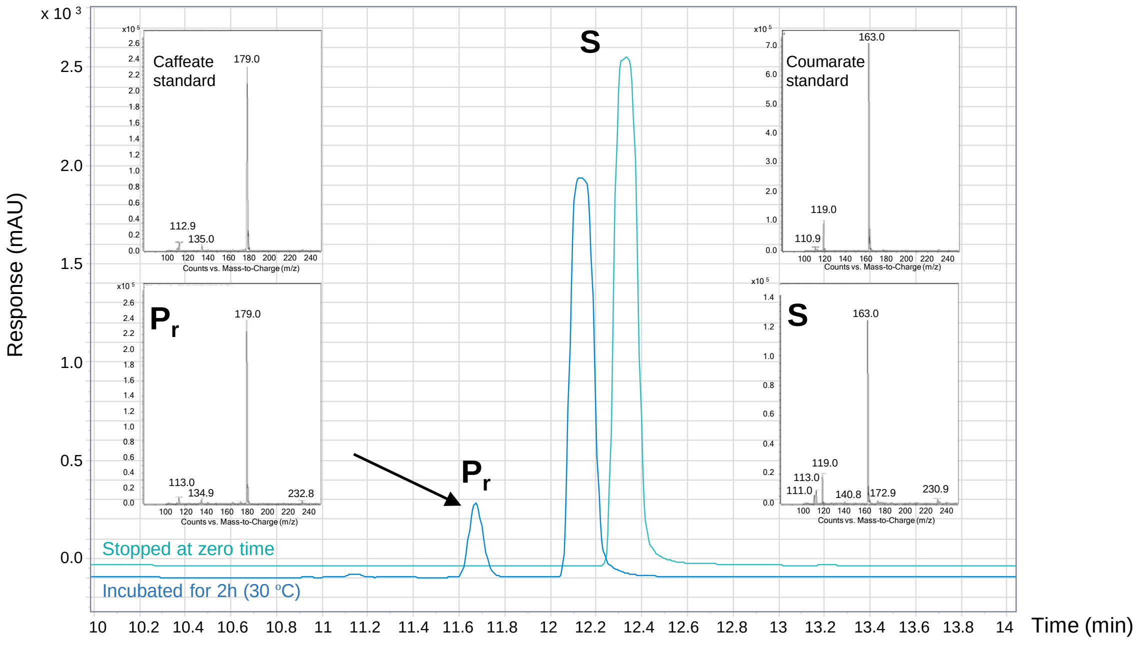

b

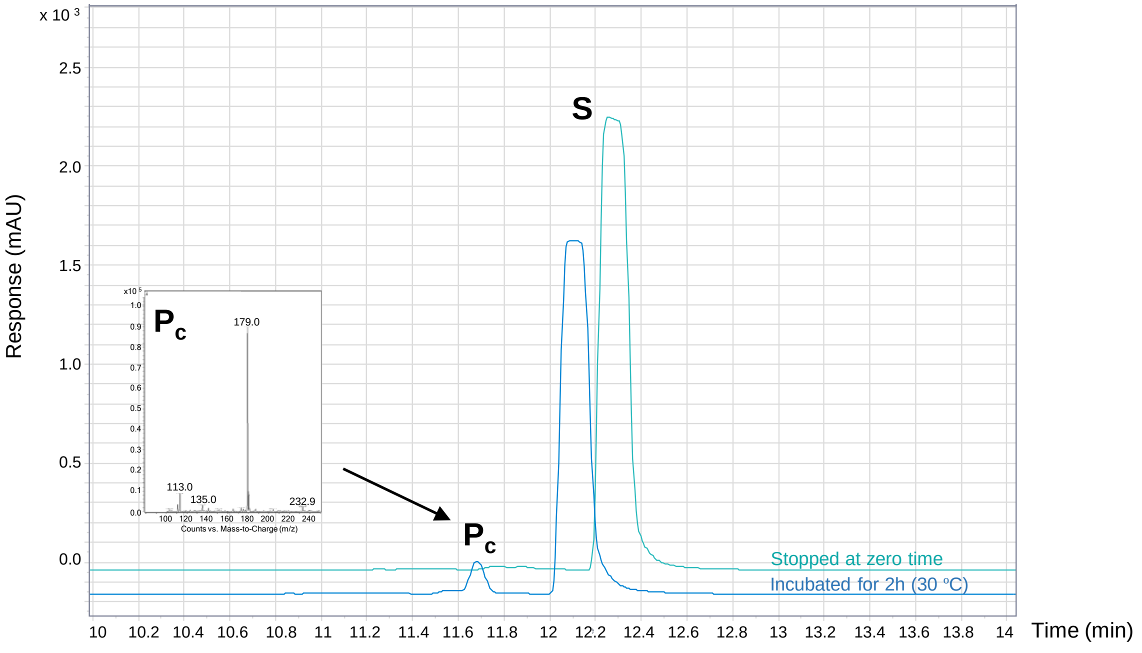

Supplementary Figure 1. LC-MS analysis of C3H *in vitro* activity assays. a, Caffeate (P_r) is detected as a product of recombinant *Brachypodium* C3H when 4-coumarate is given as substrate (S), but is not detected when the reaction is stopped at zero time with acetic acid (or incubated without ascorbate). The reaction mixture contains 10 µl of 10 mM 4-coumarate, 7.5 µl of 10 mg/ml BSA, 20 µl of 20 mM L-ascorbate, 7.5 µl of 1 M sodium phosphate buffer, 10 µl of 0.3% H_2O_2 solution, 500 ng of the hemin-reconstituted purified recombinant *Brachypodium* C3H and water up to a total volume of 100 µl. **b,** C3H activity assay using crude protein extracts from maize roots prepared for C3H identification (see Methods). Similarly, caffeate (P_c) is detected as a product of the C3H reaction using crude protein extracts. In this case, the reaction mixture contains 10 µl of 10 mM 4-coumarate, 7.5 µl of 10 mg/ml BSA, 20 µl of 20 mM L-ascorbate, 7.5 µl of 1 M sodium phosphate buffer, 15 µl of the protein crude maize root extracts (0.7 µg/µl) and water up to a total volume of 100 µl. Reactions were incubated for 2 h at 30 °C with shaking, controls were stopped at zero time with 10 µl of acetic acid. Ion masses of the standards caffeate and coumarate are shown. S, substrate 4-coumarate; P_r, caffeate as product of the C3H reaction using recombinant *Brachypodium* C3H; P_c, caffeate as product of the C3H reaction using crude protein extracts prepared from maize roots. mAU, milli-Absorbance Units at 327 nm.

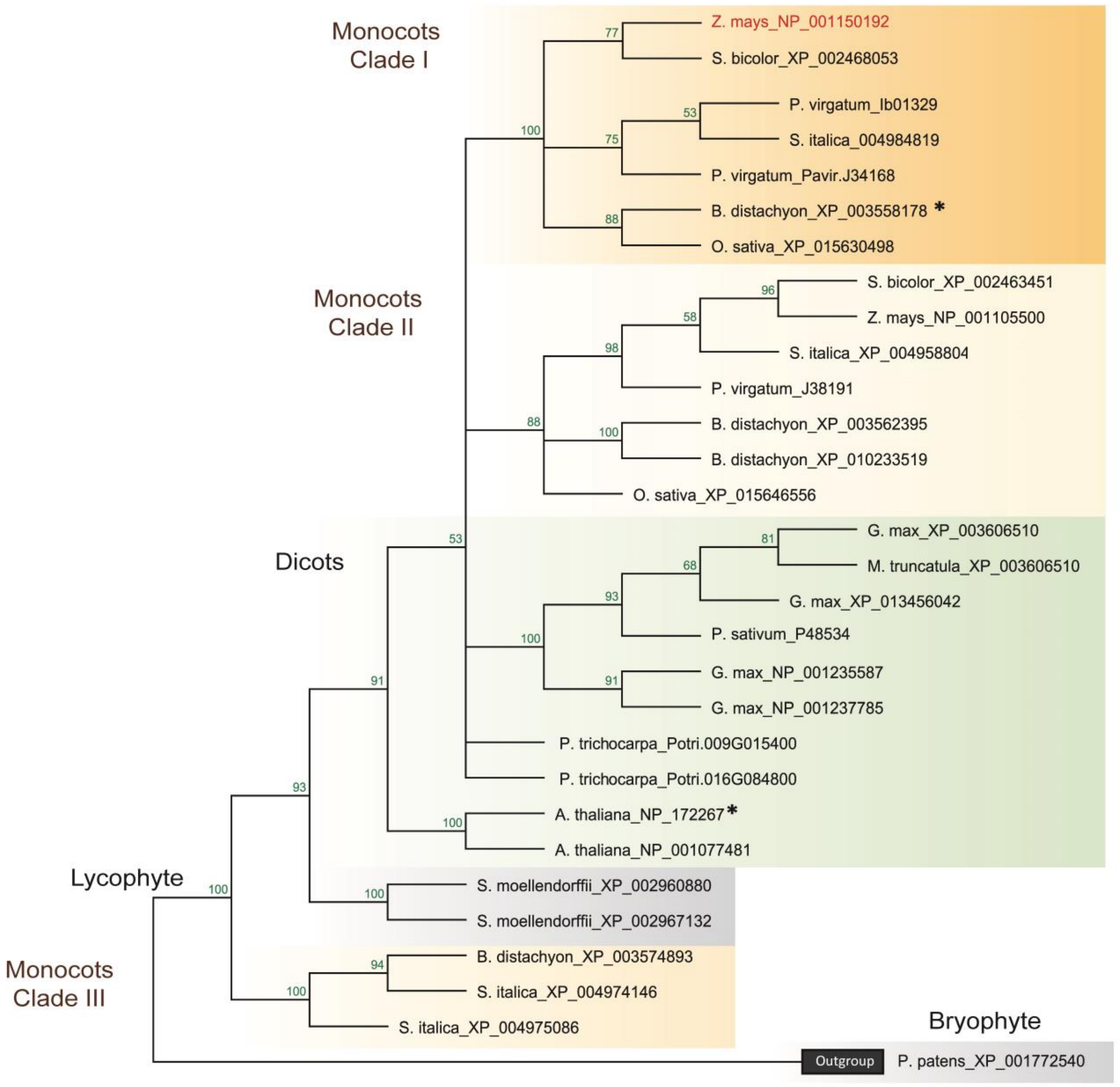

Supplementary Figure 2. Phylogenetic analysis of putative C3Hs in different plant species. Protein sequences were selected based on a standard protein BLASTP against the non-redundant protein sequence database of NCBI using maize C3H (GRMZM2G137839) as query. *Panicum virgatum* and *Populus trichocarpa* were obtained via Phytozome v12.0. *Physcomitrella patens* sequence XP_001772540 was selected as outgroup. See methods for a description of the phylogenetic tree generation. *Brachypodium* and *Arabidopsis* C3H orthologs (*****) were selected to be expressed as His-tagged fusion proteins in *E. coli* and their kinetics shown in Supplementary Table 1.

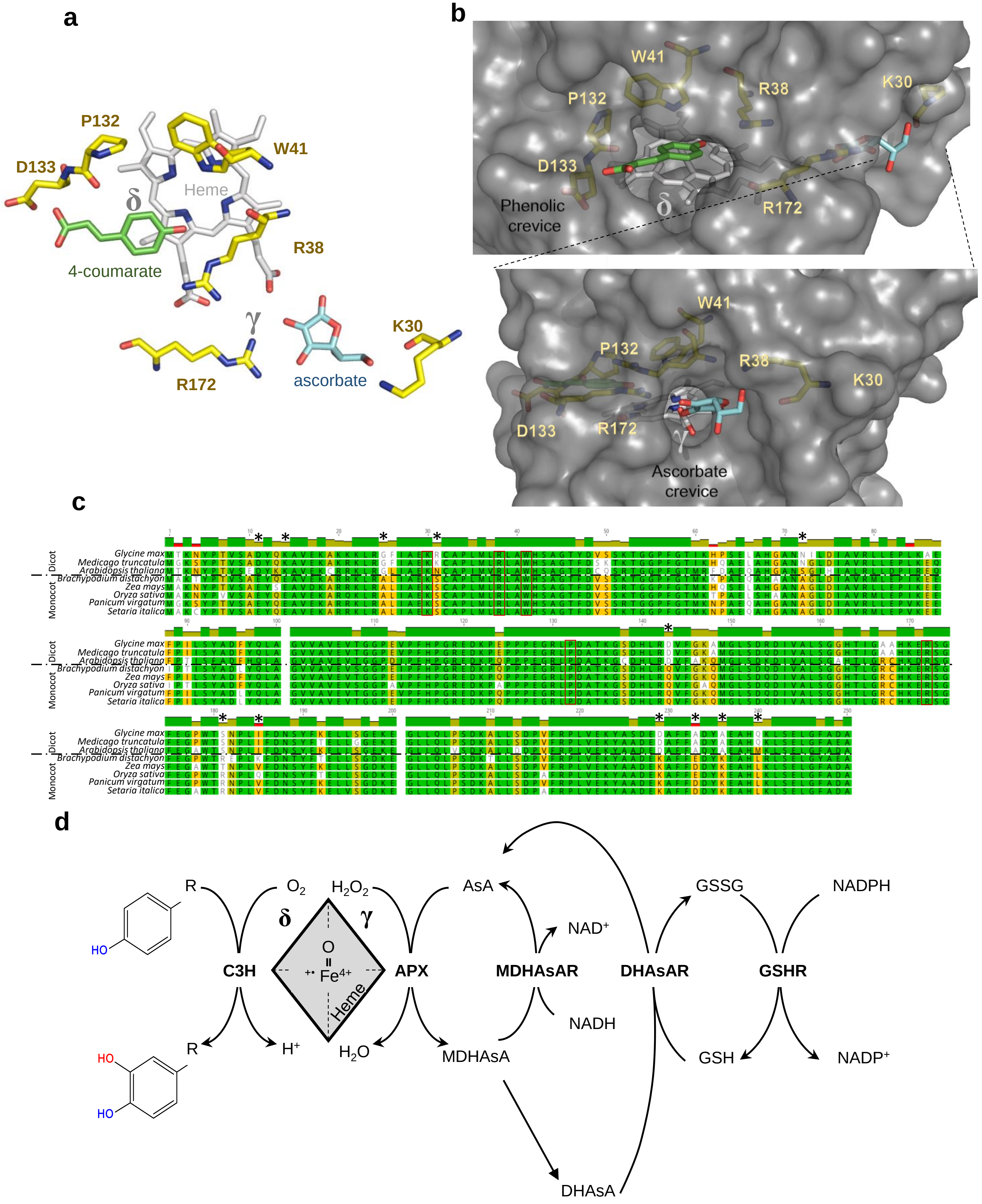

Supplementary Figure 3. Molecular modeling of C3H. a, Structure-based alignment of *Brachypodium* C3H-ascorbate (cyan) and C3H-4CA (green) complexes. The residues involved in binding of ascorbate (Lys30, Arg38 and Arg172) and 4-coumarate (Arg38, Trp41, Pro132 and Asp133) are represented with yellow sticks. Both γ- and δ-edges of the heme group are shown in grey. The crystal structure of soybean C3H bound with salicylhydroxamic acid was used as a template (PDB ID: 1V0H). **b,** Surface view of *Brachypodium* C3H viewed looking towards the phenolic (upper panel) and ascorbate (lower panel) crevices. **c,** Multiple sequence alignment of selected C3H proteins from dicot and monocot species. Asterisks indicate residues that differ in monocots and dicots. Red boxes indicate the residues above involved in substrate binding. **d,** Redox reactions involved with scavenging hydrogen peroxide coupled with the C3H hydroxylation reaction. APX, ascorbate peroxidase; AsA, ascorbate; MDHAsA, monodehydroascorbate; DHAsA, dehydroascorbate ;MDHAsAR, monodehydroascorbate reductase; DHAsAR, dehydroascorbate reductase; GSHR, glutathione reductase; GSH, reduced glutathione; GSSG, glutathione disulfide.

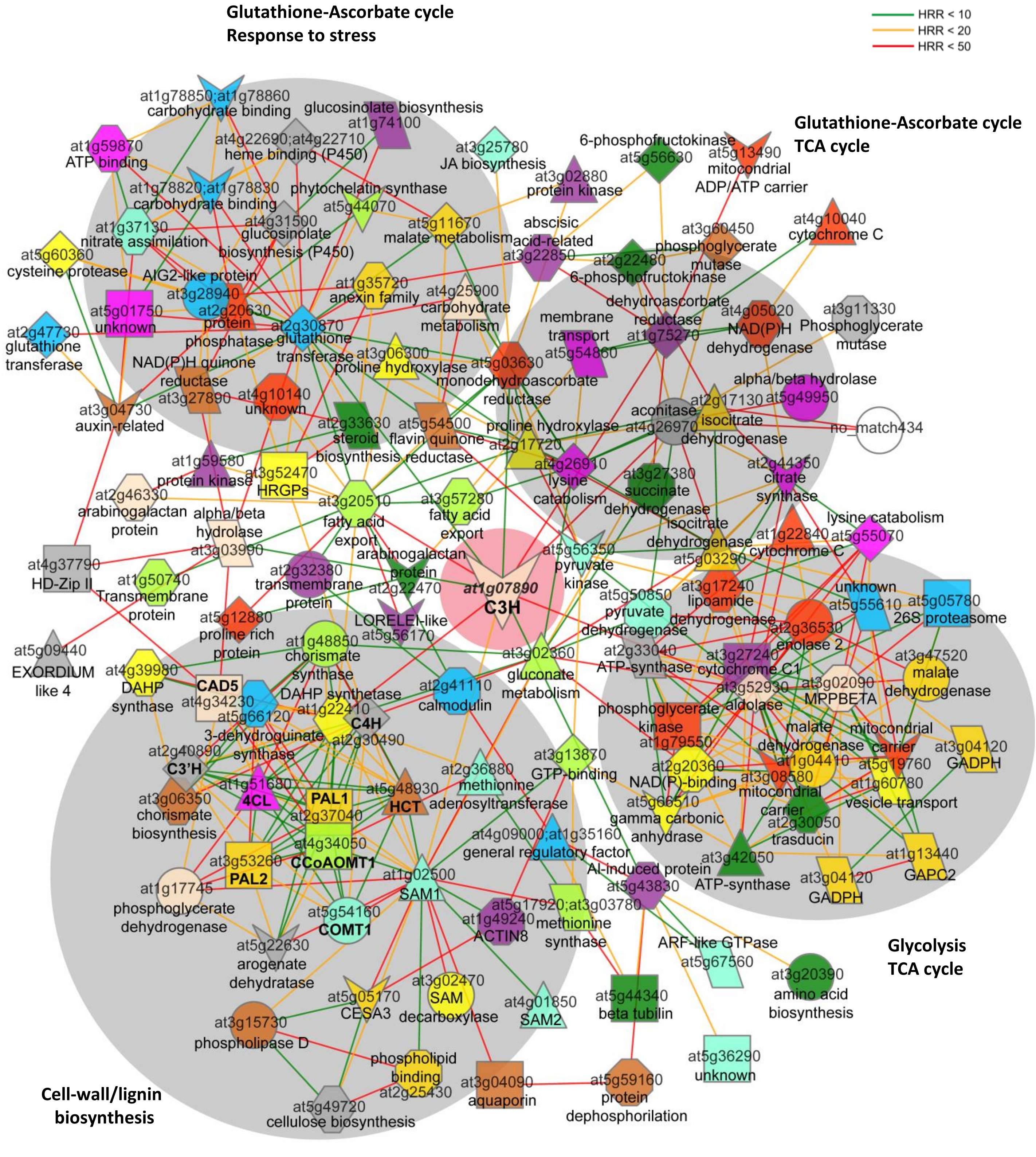

Supplementary Figure 4. Genes predicted by PlaNet to be co-expressed with *Arabidopsis C3H.* PlaNet database can be found online at: http://aranet.mpimp-golm.mpg.de The green, orange and red connectors indicate strong (HRR < 10), medium (HRR < 20) and weak (HRR < 50) strength of the coexpression, respectively. HRR, stands for highest reciprocal rank. The colored shapes of the nodes indicate label co-occurrences (same gene families or Pfam domains). See methods for a further description. The connection of C3H (At1g07890) with other lignin biosynthesis genes is through SAM1 (At1g02500) and COMT1 (At5g54160). GSH-AsA cycle, glutathione-ascorbate cycle; TCA cycle, tricarboxylic acid cycle; PAL, L-phenylalanine ammonia-lyase; C4H, cinnamate 4-hydroxylase; 4CL, 4-hydroxycinnamate:CoA ligase; C3'H, 4-coumaroyl shikimate/quinate 3-hydroxylase; CAD, cinnamyl alcohol dehydrogenase; CCoAOMT, caffeoyl-CoA 3-*O*-methyltransferase; HCT, 4-hydroxycinnamoyl CoA: shikimate/quinate hydroxycinnamoyltransferase; COMT, caffeate/5-hydroxyferulate 3-*O*-methyltransferase. *Brachypodium* C3H (Bradi1g65820) was not found to be co-expressed with any other gene in PlaNet database (see methods). We therefore searched other co-expression databases available in monocots (Supplementary Figure 5).

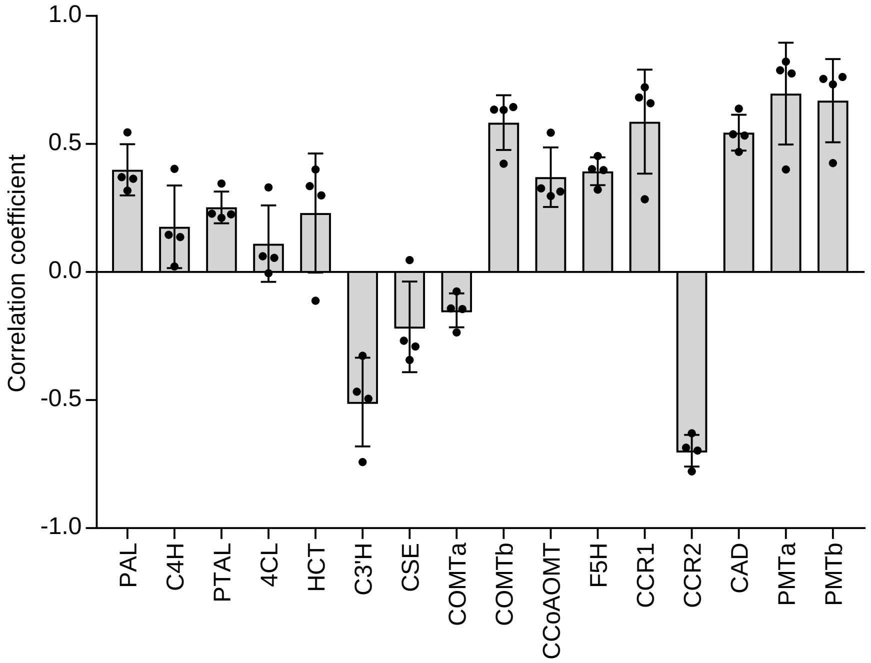

Supplementary Figure 5. Lignin genes coexpressed with *C3H* in lignifying switchgrass cell cultures. Co-expression analysis using Pearson's correlation coefficients were obtained from microarrays conducted on cell cultures in which cell wall lignification was induced by application of brassinolide at 0, 6 h, 1, 3, and 7 days and on non-induced samples at 1 and 7 days. See methods for a further description. Four C3H target sequences were used for the analysis: AP13CTG09405_s_at, KanlowCTG05802_s_at, KanlowCTG21274_at, and KanlowCTG24102_s_at. Target sequences for other lignin genes: PAL (KanlowCTG00211_s_at), C4H (KanlowCTG11894_s_at), PTAL (KanlowCTG00004_s_at), 4CL (KanlowCTG00833RC_s_at), HCT (AP13CTG44530_s_at), C3H (AP13ITG41630_at), CSE (AP13ITG63270_at), COMTa (KanlowCTG00989_s_at), COMTb (AP13ITG62564_at), CCoAOMT (KanlowCTG00900_s_at), F5H (AP13ITG56842_at), CCR1 (KanlowCTG19403_s_at), CCR2 (AP13ITG69021_s_at), CAD (VS16ITG06742_s_at), PMTa (AP13ITG57008_at), and PMTb (AP13CTG16683_at). Except for PMT (p-Coumaroyl-CoA:monolignol transferase), CCR (Cinnamoyl-CoA reductase) and F5H (ferulate 5-hydroxylase) all other abbreviations are described in Figure 1 and Supplementary Figure 4. Error bars indicate mean ± SD. n= 4.

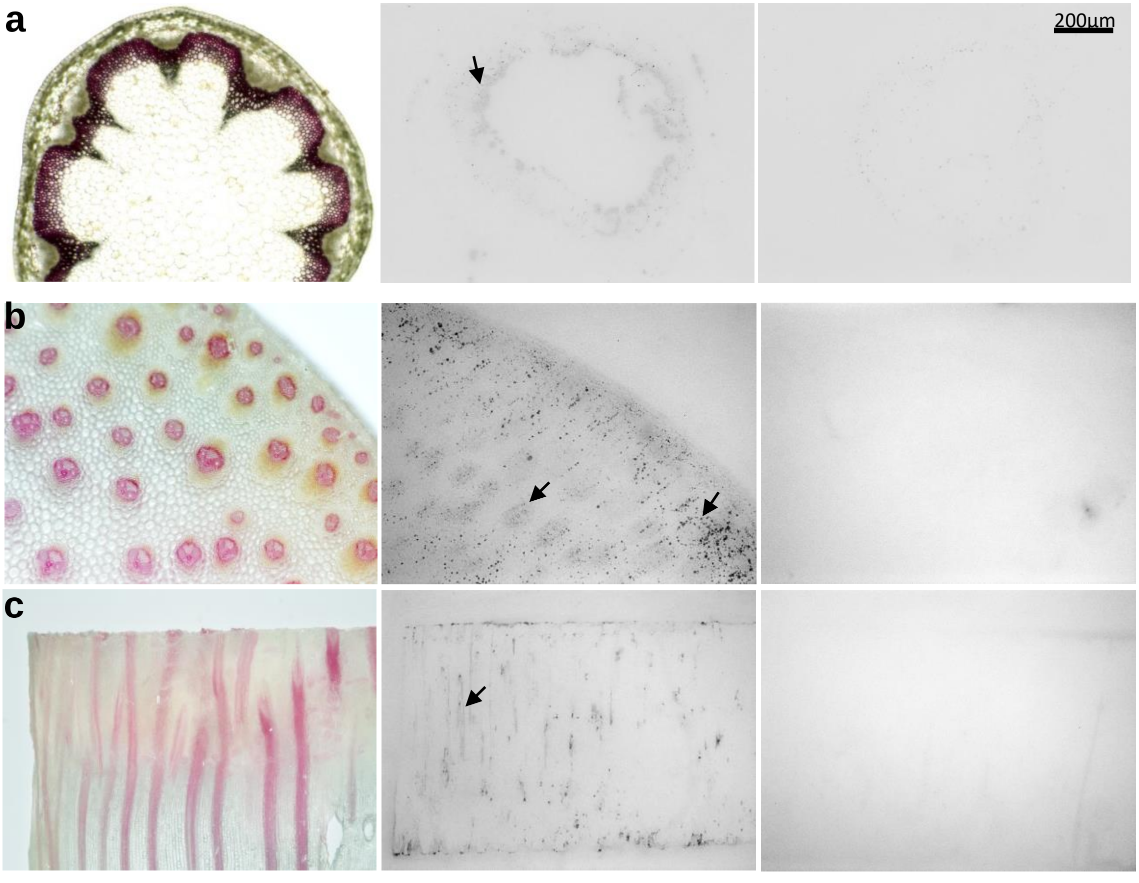

Supplementary Figure 6. Immunolocalization of C3H in *Arabidopsis* and maize stems by tissue printing. Left panels, microtome-cut sections of stems stained with phloroglucinol-HCl, the red coloration reflects cinnamaldehyde end-groups of lignin. Middle panels, sections printed onto nitrocellulose and developed using anti-C3H/APX1 polyclonal antibodies. Right panels, negative controls using pre-immune serum. **a,** *Arabidopsis thaliana* transversal section; **b,** *Zea mays* transversal section; **c,** *Zea mays* longitudinal section.

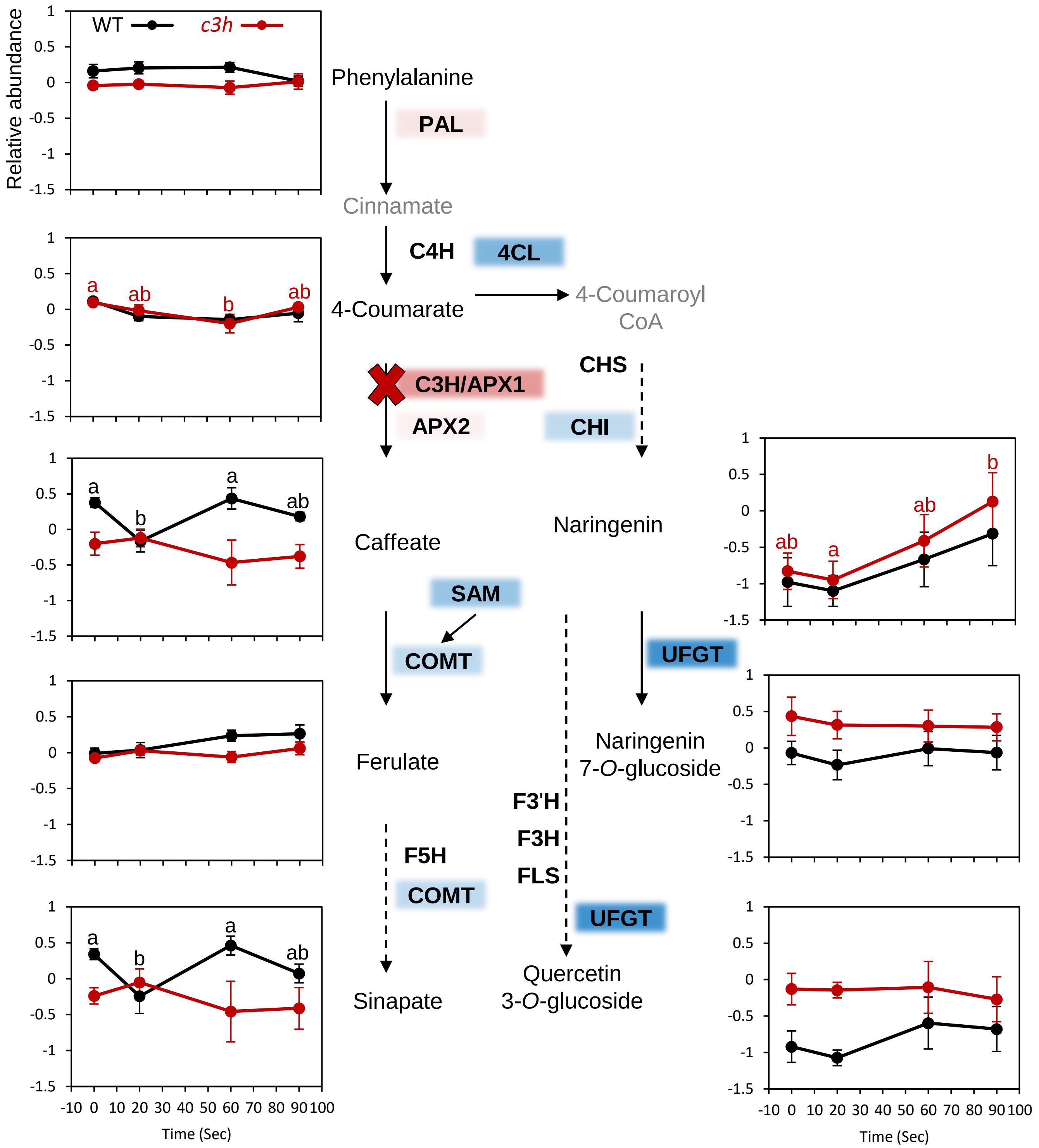

Supplementary Figure 7. Metabolomic and transcriptomic shifts in *Arabidopsis c3h1* mutants after light stress. Relative increase and decrease of transcript and metabolite abundances in 18-21-day-old leaves from *c3h* mutants compared with Col-0 wild-type plants mapped on the early steps of the lignin/flavonoid pathways. Levels of mRNA transcripts obtained from Pnueli *et al.*[1] are indicated in colored boxes (red and blue represents increased and decreased transcript abundance, respectively). Corresponding locus IDs and fold-change expression levels relative to wild-type: PAL: At5g042 (0.6); 4CL: At1g20490 (3.4), At3g21230 (1.3); C3H/APX1: At1g07890 (0.04); APX2: At3g09640 (0.90); COMT: At5g54160 (1.7); SAM: At3g44870 (3.0); CHI: At2g26310 (1.3); UFGT: At1g30530 (7.6), At4g34135 (7.7). Differences in metabolites are indicated in line charts: *c3h1* mutants are shown as red lines and wild-type plants as black lines. Metabolites were measured at four different time points (0, 20, 60 and 90 seconds) as described in methods. Intermediates below detection limit are shown in grey. Units are relative abundances (natural log of the scaled intensity). Error bars indicate mean ± SEM, n = 5, and letters denote significant differences between means of different time points in wild-type (black) and mutant (red) at $P < 0.05$ according to a one-way ANOVA (Duncan's test) analysis. Abbreviations: SAM, S-adenosyl-L-methionine methyltransferase; CHS, chalcone synthase; CHI, chalcone isomerase; UFGT, UDP-glucose flavonoid 3-*O*- and 7-*O*-glucosyl transferase; F3'H, flavonoid 3'-monooxygenase; F3H, flavanone-3-hydroxylase; FLS, flavonol synthase. Dashed arrows indicate multiple biosynthetic steps. Other abbreviations are described in Figure 1 and Supplementary Figure 4.

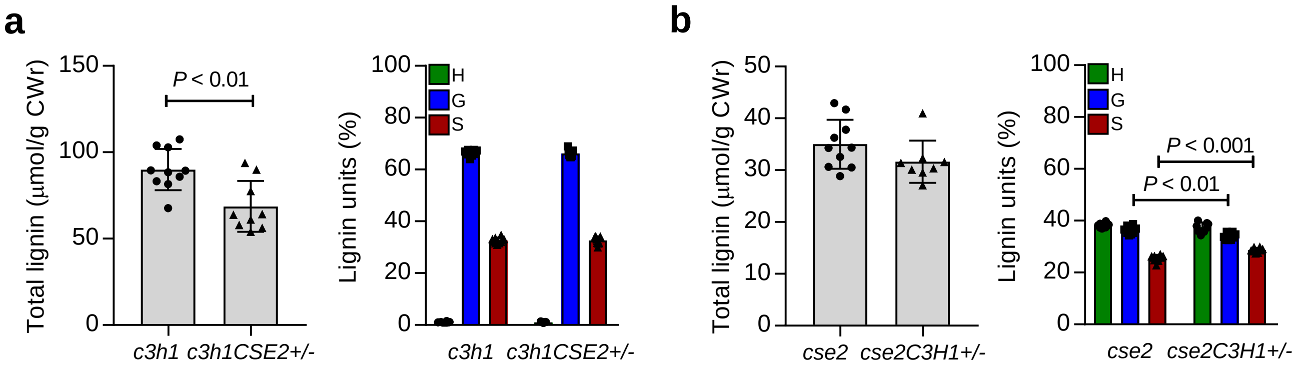

Supplementary Figure 8. Lignin content and composition of *Arabidopsis c3h1CSE2+/-* and *cse2C3H1+/-* mutants determined by thioacidolysis. The double *c3h1:cse2* knockout mutants in *Arabidopsis* are embryo lethal (Figure 4c-e). **a,** Total lignin amount and composition of *c3h1CSE2+/-* and *c3h1* mutant controls. **b,** Total lignin amount and composition of *c3h1CSE2+/-* and *c3h1* mutant controls. Error bars indicate mean ± SD, two-sided unpaired t-test. CWr, cell wall residue. n = 8-10

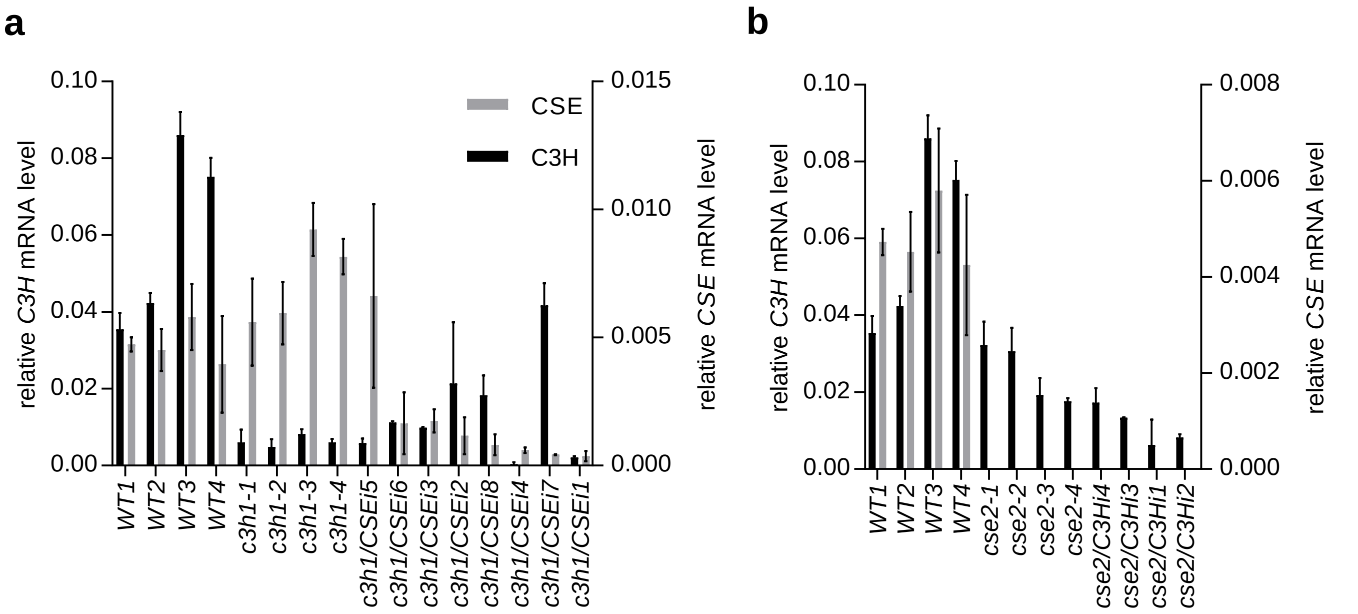

Supplementary Figure 9. Transcript levels of *cse* and *c3h* in *Arabidopsis* RNAi lines compared to controls. CSE and C3H transcripts in *c3h1/CSE* RNAi lines (**a**) and *cse2/C3H* RNAi lines (**b**) and WT, *c3h1* and *cse2* mutants controls as defined by qPCR. Error bars indicate mean ± SD.

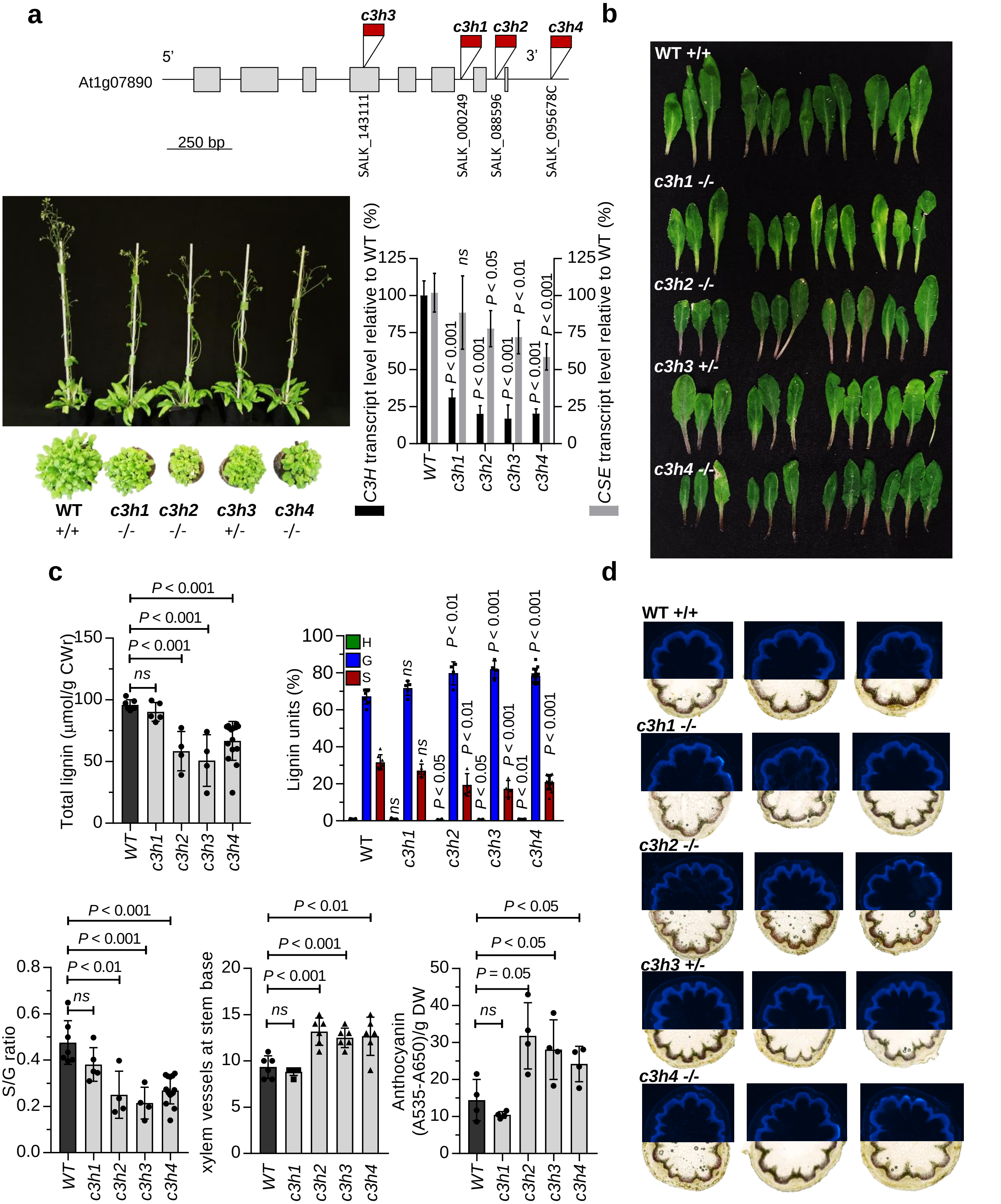

Supplementary Figure 10. Characterization of additional *c3h* mutant alleles in *Arabidopsis*. a, Position of the T-DNA insertion in all SALK *c3h* mutant lines available in *Arabidopsis* showing their growth phenotype for 2-month-old plants grown in individual pots and 25-day-old plants grown on peat pellets and *c3h* and *cse* expression levels as defined by qPCR, obtained using cDNAs from stems of 4-8 individual 2-month-old plants for each genotype. No *c3h3* homozygous mutants (the only mutant available with the T-DNA insertion in the exon) were obtained from over 125 seeds received from the stock center, and so heterozygous *c3h3*+/– plants were characterized. We failed to find *c3h3* homozygous mutants also in a screen of ~100 T2-generation *c3h3*+/– plants. **b,** Leaf phenotype at harvest (2-month-old plants) of wild-type controls and all *c3h* mutants characterized. **c,** Lignin levels, relative monolignol composition and S/G ratios determined by thioacidolysis, as well as number of xylem vessels at stem base and anthocyanin content for wild-type controls and all *c3h* mutants. **d,** Transverse stem sections (UV-autofluorescence and phloroglucinol-HCl staining) of wild-type controls and all *c3h* mutants. Error bars indicate mean ± SD. n = 3-5 (panel **a**), Data points for all biological replicates are shown in panel **c**.

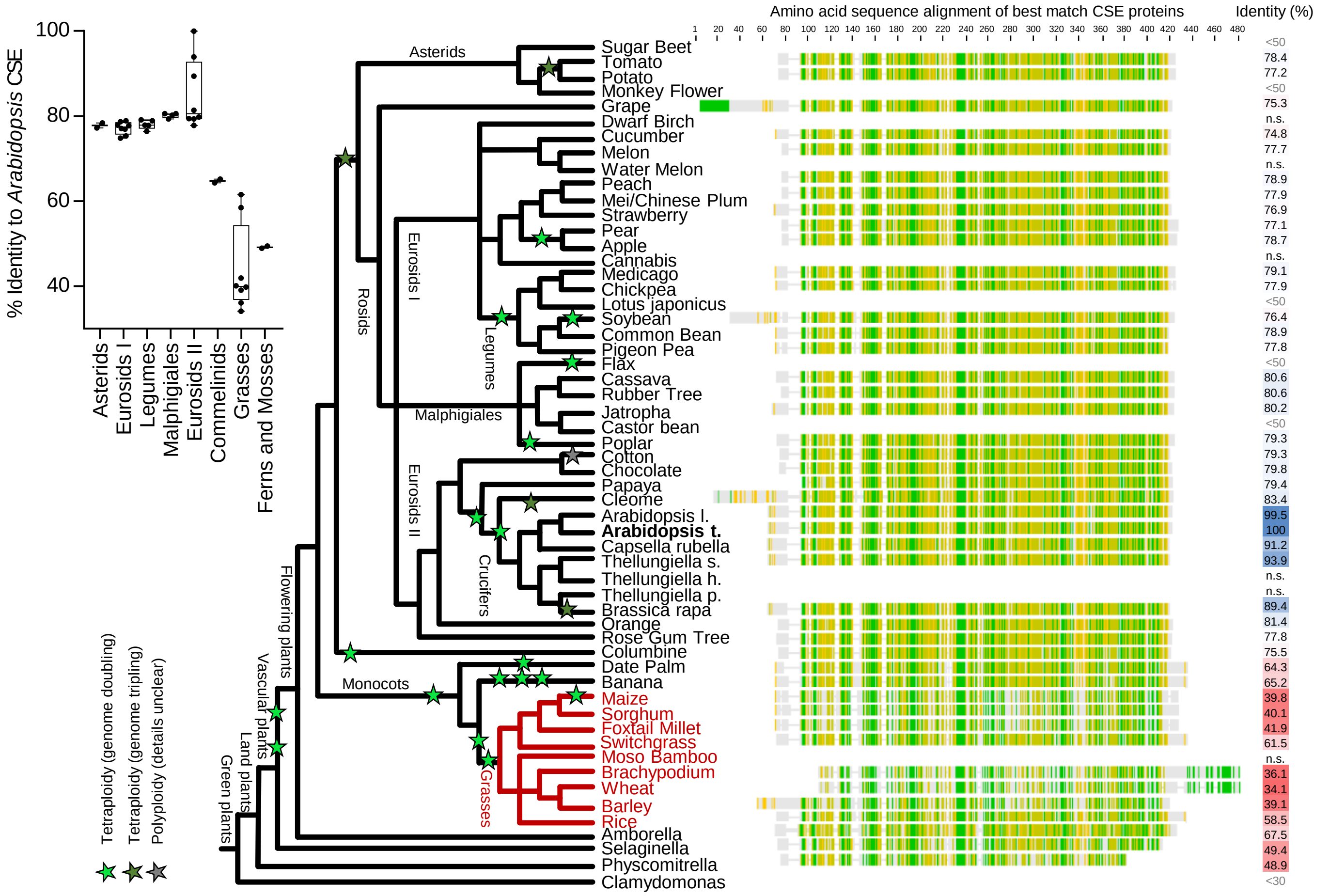

Supplementary Figure 11. The lack of caffeoyl shikimate esterase (CSE) homologs is phylogenetically related to monocot plants. Amino acid sequence alignment of best match CSE proteins from multiple plants species and percent of identity to the reference *Arabidopsis thaliana* CSE[2]. Amino acid sequences were obtained from NCBI GeneBank database for the following species: *Solanum lycopersicum* (taxid:4081), *Solanum tuberosum* (taxid:4113), *Mimulus guttatus* (taxid:4155), *Vitis vinifera* (taxid:29760), *Betula nana* (taxid:216990), *Cucumis sativus* (taxid:3659), *Cucumis melo* (taxid:3656), *Citrullus lanatus* (taxid:3654), *Prunus persica* (taxid:3760), *Prunus mume* (taxid:102107), *Fragaria vesca* (taxid:57918), *Pyrus communis* (taxid:23211), *Malus domestica* (taxid:3750), *Cannabis sativa* (taxid:3483), *Medicago truncatula* (taxid:3880), *Cicer arietinum* (taxid:3827*), Lotus japonicus* (taxid:34305), *Glycine max* (taxid:3847), *Phaseolus vulgaris* (taxid:3885), *Cajanus cajan* (taxid:3821), *Linum usitatissimum* (taxid:4006), *Manihot esculenta* (taxid:3983), *Hevea brasiliensis* (taxid:3981), *Jatropha curcas* (taxid:180498), *Ricinus communis* (taxid:3988), *Populus trichocarpa* (taxid:3694), *Gossypium hirsutum* (taxid:3635), *Theobroma cacao* (taxid:3641), *Carica papaya* (taxid:3649), *Arabidopsis thaliana* (taxid:3702), *Arabidopsis lyrata* (taxid:59689), Capsella rubella (taxid:81985),*Thellungiella salsuginea* (taxid:72664), *Thellungiella parvula* (taxid:98039), *Brassica rapa* (taxid:3711), *Citrus sinensis* (taxid:2711), *Eucalyptus grandis* (taxid:71139), *Aquilegia coerulea* (taxid:218851), *Phoenix dactylifera* (taxid:42345), *Musa acuminata* (taxid:4641), *Zea mays* (taxid:4577), *Sorghum bicolor* (taxid:4558), *Setaria italica* (taxid:4555), *Panicum virgatum* (taxid:38727), *Phyllostachys edulis* (taxid:38705), *Brachypodium distachyon* (taxid:15368), *Triticum aestivum* (taxid:4565), *Hordeum vulgare subsp. vulgare* (taxid:112509), *Oryza sativa subsp. indica* (taxid:39946), *Amborella trichopoda* (taxid:13333), *Selaginella moellendorffii* (taxid:88036), *Physcomitrella patens* (taxid:3218) and *Chlamydomonas reinhardtii* (taxid:3055). Species with *CSE* genes with identity to Arabidopsis *CSE* below 50% were not included in the multiple sequence alignment, n.s. not significant identity, even when searched in nearby species of the same genus. Boxplot: center line, median; box limits, upper and lower quartiles; whiskers, min to max range.

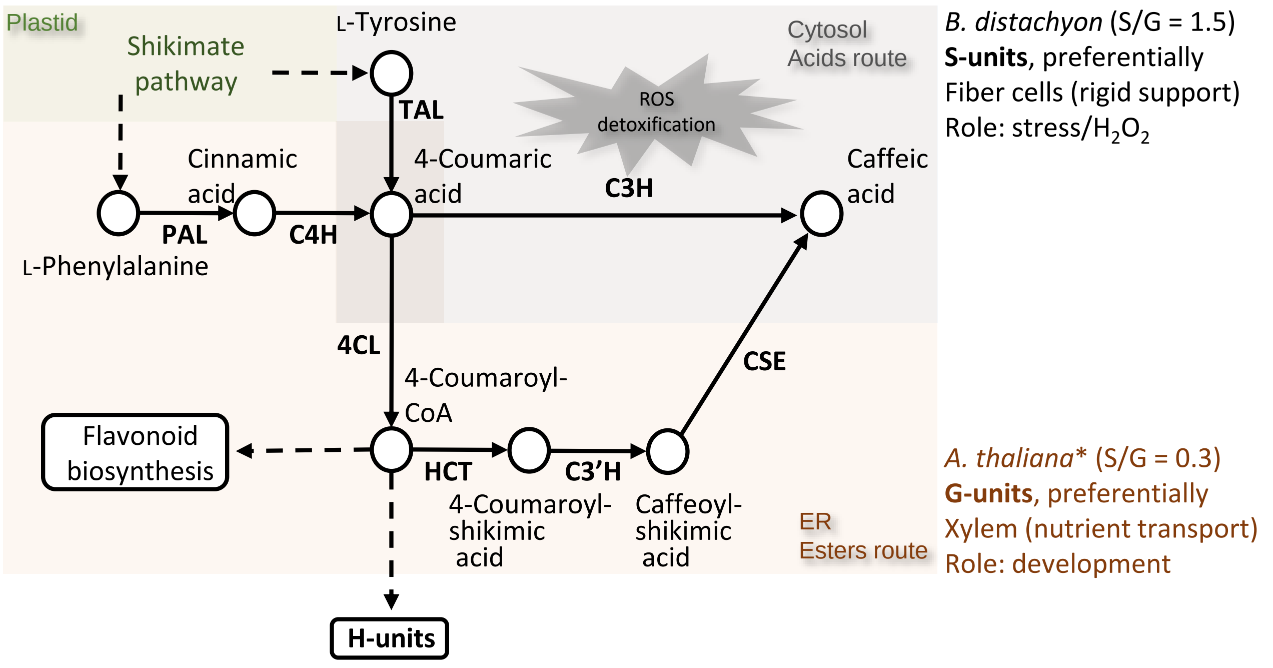

Supplementary Figure 12. A simplified model for the first steps of lignin biosynthesis in angiosperms. Dotted arrows, multiple enzymatic steps. The routes to caffeoyl derivatives, and therefore to G- and S-units of lignin, involves C3'H (via the ER-localized esters pathway) or C3H/APX1 (through the cytosol-localized acids pathway). Abbreviations: TAL, multifunctional L-phenylalanine/L-tyrosine/L-dopa ammonia-lyase; PAL, L-phenylalanine ammonia-lyase; C4H, cinnamate 4-hydroxylase; C3H, 4-coumarate 3-hydroxylase; 4CL, 4-hydroxycinnamate:CoA ligase; HCT, 4-hydroxycinnamoyl CoA: shikimate/quinate hydroxycinnamoyltransferase; C3'H, 4-coumaroyl shikimate/quinate 3-hydroxylase; CSE, caffeoyl shikimate esterase; ER, endoplasmic reticulum.

Supplementary Table 1. Enzyme kinetics of C3H. Kinetic data for ascorbate peroxidase (APX), 4-coumarate 3-hydroxylase (C3H), and tyrosine hydroxylase (T3H) activity for the recombinant hemin-reconstituted proteins from *Arabidopsis* (At1g07890) and *Brachypodium* (Bradi1g65820) plotted according to the Hill equations. n is the Hill coefficient. Under the conditions tested, C3H does not convert 4-coumaroyl shikimate, 4-coumaroyl CoA, 4-coumaraldehyde or 4-coumaryl alcohol into their corresponding caffeoyl derivatives. Data are average values from three replicates ± SD.

**R*eaction* (substrate → product) | *Species***	**K_m (µM)**	**K_{cat} (s^{-1})**	**K_{cat}/K_m ($\mu M^{-1}\ s^{-1}$)**	**n**
APX (ascorbate → dehydroascorbate) | *Brachypodium*	342 ± 57	131	0.38	1.9
APX (ascorbate → dehydroascorbate) | *Arabidopsis*	299 ± 63	181	0.60	2.6
C3H (4-coumarate → caffeate) | *Brachypodium*	604 ± 138	463	0.77	1.6
C3H (4-coumarate → caffeate) | *Arabidopsis*	708 ± 154	492	0.70	0.9
T3H (tyrosine → levodopa) | *Brachypodium*	964 ± 191	732	0.76	1.3
T3H (tyrosine → levodopa) | *Arabidopsis*	653 ± 127	599	0.92	1.7

Supplementary Table 2. Segregation ratios of *Arabidopsis c3h1CSE2+/–* and *cse2C3H1+/–* mutants. Seeds were analyzed from 8 to 18 siliques in each genotype. The genotypes of each plant were confirmed by PCR before analysis. For the controls (WT, *cse2* and *c3h1* mutants) three individual plants were used, for *c3h1CSE2+/–* four plants and for *cse2C3H1+/–* five plants.

**Genotype**	**Seeds/silique (average)**	**Normal seeds (total)**	**Aborted seeds (total)**	**Normal/Aborted**
WT	40.6 ± 6.5 (n = 8)	325	13	25:1
c3h1	34.1 ± 9.5 (n = 9)	307	13	24:1
cse2	30 ± 11.5 (n = 11)	330	28	12:1
c3h1CSE2+/–	27.5 ± 8.0 (n = 13)	357	68	5:1
cse2C3H1+/–	20.7 ± 8.5 (n = 18)	393	190	2:1

Supplementary Table 3. Lignin amount determined by the acetyl bromide method. Acetyl bromide lignin content for *c3h* mutants and both wild-type and T-DNA control line JJ22251 (*apx3*) in *Brachypodium*, and for *cse2* mutant/C3H-RNAi (*cse2:C3Hi*) and c3h1 mutant/CSE-RNAi (*c3h1:CSEi*) lines compared to their respective T-DNA mutant only controls and wild-type plants in *Arabidopsis*. Data are average values from three independent biological replicates ± SD. *0.05>P; two-sided unpaired t test.

**Genotype**	**Acetyl bromide lignin (mg/gCWr)**
Brachypodium distachyon	
WT	240.7 ± 15.2
apx3	233.6 ± 4.6
c3h	179.1 ± 4.9*
Arabidopsis thaliana	
WT	216.0 ± 5.0
c3h1	213.7 ± 7.7
c3h1:CSEi	169.7 ± 3.3*
cse2	107.8 ± 7.9
cse2:C3Hi	82.0 ± 6.0*

Supplementary Table 4. Apparent kinetics of maize C3H and 4CL. Maize (*Zea mays*) root crude protein extracts prepared during the purification experiments were used to compare the relative apparent kinetic constants of C3H with its competing reaction 4-coumarate:CoA ligase (4CL). Data are average values from three replicates ± SD.

***Species* | *reaction* (substrate → product)**	**K_m (µM)**	**Specific activity (pkat/mg protein)**
Zea mays | *C3H* (4-coumarate → caffeate)	97 ± 26.7	76.1 ± 5.5
Zea mays | *4CL* (4-coumarate → 4-coumaroyl CoA)	20 ± 5.8	42.6 ± 4.8

Supplementary References

1. Pnueli, L., Liang, H., Rozenberg, M. & Mittler, R. Growth suppression, altered stomatal responses, and augmented induction of heat shock proteins in cytosolic ascorbate peroxidase (Apx1) - deficient *Arabidopsis* plants. *Plant J.* **34**, 187–203 (2003).

2. Vanholme, R. et al.Caffeoyl shikimate esterase (CSE) is an enzyme in the lignin biosynthetic pathway in *Arabidopsis*. *Science* **341**, 1103–1106 (2013).
