# Supplementary material for: 4-Coumarate 3-hydroxylase in the lignin biosynthesis pathway is a cytosolic ascorbate peroxidase
Source: Nat Commun. 2019 Apr 30;10:1994. doi: 10.1038/s41467-019-10082-7 (PMC6491607; doi:10.1038/s41467-019-10082-7)
Supplement: Supplementary file 2 — Reporting Summary [file 41467_2019_10082_MOESM2_ESM.pdf]

natureresearch

Corresponding author(s): DBPR_NCOMMS-18-26991A

Last updated by author(s): Mar 25, 2019

Reporting Summary

Nature Research wishes to improve the reproducibility of the work that we publish. This form provides structure for consistency and transparency in reporting. For further information on Nature Research policies, see Authors & Referees and the Editorial Policy Checklist.

Statistics

For all statistical analyses, confirm that the following items are present in the figure legend, table legend, main text, or Methods section.

n/a	Confirmed	
☐	☒	The exact sample size (*n*) for each experimental group/condition, given as a discrete number and unit of measurement
☒	☐	A statement on whether measurements were taken from distinct samples or whether the same sample was measured repeatedly
☐	☒	The statistical test(s) used AND whether they are one- or two-sided<br>*Only common tests should be described solely by name; describe more complex techniques in the Methods section.*
☒	☐	A description of all covariates tested
☒	☐	A description of any assumptions or corrections, such as tests of normality and adjustment for multiple comparisons
☐	☒	A full description of the statistical parameters including central tendency (e.g. means) or other basic estimates (e.g. regression coefficient) AND variation (e.g. standard deviation) or associated estimates of uncertainty (e.g. confidence intervals)
☐	☒	For null hypothesis testing, the test statistic (e.g. *F*, *t*, *r*) with confidence intervals, effect sizes, degrees of freedom and *P* value noted<br>*Give P values as exact values whenever suitable.*
☒	☐	For Bayesian analysis, information on the choice of priors and Markov chain Monte Carlo settings
☒	☐	For hierarchical and complex designs, identification of the appropriate level for tests and full reporting of outcomes
☒	☐	Estimates of effect sizes (e.g. Cohen's *d*, Pearson's *r*), indicating how they were calculated

Our web collection on statistics for biologists contains articles on many of the points above.

Software and code

Policy information about availability of computer code

Data collection	Light microscope images were taken with an EVOS™ XL Core Imaging System (Thermo Fisher). The images showing lignin autofluorescence were captured with an EVOS FL Cell Imaging System (Thermo Fisher) equipped with a DAPI led light cube (excitation: 357/44 nm; emission: 447/60 nm). Images for the seed phenotype characterization in Arabidopsis were taken with an AxioCam MRc 5 camera attached to a Zeiss Axio Zoom.V16 stereo zoom microscope.
Data analysis	For statistical analyses, Microsoft Excel 2010 and GraphPad Prism 7 softwares were used.

For manuscripts utilizing custom algorithms or software that are central to the research but not yet described in published literature, software must be made available to editors/reviewers. We strongly encourage code deposition in a community repository (e.g. GitHub). See the Nature Research guidelines for submitting code & software for further information.

Data

Policy information about availability of data

All manuscripts must include a data availability statement. This statement should provide the following information, where applicable:

- Accession codes, unique identifiers, or web links for publicly available datasets
- A list of figures that have associated raw data
- A description of any restrictions on data availability

The source data underlying Figs 2a, 2b, 2d, 3b, 3d, 3f, 4b, 4c, 4d, 4g, 4i, 5a, 5d, Supplementary Figs 5, 6, 7, 8a, 8b and Supplementary Tables 1,2,3, and 4 are provided as a Source Data file.

Field-specific reporting

Please select the one below that is the best fit for your research. If you are not sure, read the appropriate sections before making your selection.

☒ Life sciences ☐ Behavioural & social sciences ☐ Ecological, evolutionary & environmental sciences

For a reference copy of the document with all sections, see nature.com/documents/nr-reporting-summary-flat.pdf

Life sciences study design

All studies must disclose on these points even when the disclosure is negative.

Sample size	Sample sizes were chosen on the basis of preliminary experiments (and previous publications) to provide sufficient power for statistical comparison. Power analysis was not performed a priori.
Data exclusions	No data were excluded from this study.
Replication	All replicates are reported in the manuscript.
Randomization	Experiments described here were not randomized.
Blinding	Complete blinding for data collection was not necessary. However, the individual plants constituting technical replicates were randomly numbered to perform partially-blinded experiments.

Reporting for specific materials, systems and methods

We require information from authors about some types of materials, experimental systems and methods used in many studies. Here, indicate whether each material, system or method listed is relevant to your study. If you are not sure if a list item applies to your research, read the appropriate section before selecting a response.

Materials & experimental systems

n/a	Involved in the study	
☐	☒	Antibodies
☒	☐	Eukaryotic cell lines
☒	☐	Palaeontology
☒	☐	Animals and other organisms
☒	☐	Human research participants
☒	☐	Clinical data

Methods

n/a	Involved in the study	
☒	☐	ChIP-seq
☒	☐	Flow cytometry
☒	☐	MRI-based neuroimaging

Antibodies

Antibodies used	For immunoblot analysis a polyclonal antibody against C3H from pea (Pisum sativum) produced in rabbit (Plant Physiology 1991; 97:962-968) and a polyclonal horseradish peroxidase conjugated goat anti-rabbit IgG (Catalog # 65-6120, Sigma-Aldrich) were used as primary (1:2000) and the secondary antibody (1:50000), respectively.
Validation	Antibodies raised against the cytosolic ascorbate peroxidase from pea did not cross-react with either protein extracts obtained from intact pea chloroplasts or horseradish peroxidase. and have been validated in several previous works:<br>Plant Journal 34, 187–203 (2003); Plant Cell 17, 268–281 (2005)
